# Supplementary material for: Perceived losses of scientific integrity under the Trump administration: A survey of federal scientists
Source: PLoS One. 2020 Apr 23;15(4):e0231929. doi: 10.1371/journal.pone.0231929 (PMC7179855; doi:10.1371/journal.pone.0231929)
Supplement: S1 Appendix — (DOCX) [file pone.0231929.s001.docx]

**Supporting Information**

Perceived Losses of Scientific Integrity under the Trump Administration: A Survey of Federal Scientists

Gretchen T. Goldman, Jacob M. Carter, Yun Wang, Janice M. Larson

# APPENDIX A. Survey of Federal Scientists Response Rates, 2018.

| **Agency** | **Eligible Sample** | **Completed Surveys** | **Response Rate** |
| --- | --- | --- | --- |
| ARS | 2426 | 210 | 8.66% |
| BOEM | 407 | 63 | 15.48% |
| BSEE | 309 | 32 | 10.36% |
| CDC | 10531 | 605 | 5.74% |
| EERE | 897 | 44 | 4.91% |
| EPA | 14856 | 449 | 3.02% |
| ERS | 307 | 23 | 7.49% |
| FDA | 9378 | 354 | 3.77% |
| NASS | 752 | 20 | 2.66% |
| NIFA | 138 | 5 | 3.62% |
| NOAA | 11195 | 1158 | 10.34% |
| FWS | 4383 | 360 | 8.21% |
| USGS | 2934 | 561 | 19.12% |
| US CENSUS BUREAU | 2947 | 64 | 2.17% |
| US National Park Service | 1274 | 231 | 18.13% |
| NHTSA | 392 | 24 | 6.12% |
| Other Agency* |  | 8 | N/A |
| **Totals** | **63126** | **4211** | **6.67%** |

* Cases recorded as Other Agency were sampled in the above agencies but recorded their agency as “Other" in their completed survey.
