# Supplementary material for: Perceived losses of scientific integrity under the Trump administration: A survey of federal scientists
Source: PLoS One. 2020 Apr 23;15(4):e0231929. doi: 10.1371/journal.pone.0231929 (PMC7179855; doi:10.1371/journal.pone.0231929)
Supplement: S2 Appendix — (DOCX) [file pone.0231929.s002.docx]

# APPENDIX B. Survey E-Mail Invitation

E-mail Sending Name: Iowa State University

E-mail Sending Address: [isu-cssm@qualtrics-research.com](mailto:isu-cssm@qualtrics-research.com) Subject Line: UCS Scientific Integrity Study

Dear Federal Scientist,

Researchers at the Iowa State University Center for Survey Statistics and Methodology (CSSM) and the Union of Concerned Scientists (UCS) are conducting a survey of over 63,000 science professionals from 16 science-based US government agencies. Your agency is included in this study. You were chosen to receive a survey invitation because your office and/or job title indicated your work deals with science in some capacity.

With your help, we hope to better understand the role of science and scientific integrity at your agency and the contributions of scientists in fulfilling their agency’s science-based mission during the past year.

**For quality control, you will need the following access code to complete the survey: XXXXXXXX**. We assure you that your privacy is of the utmost concern to us. Any personally identifying information will be destroyed immediately when your survey responses are submitted.

The survey is available to you in three ways.

Option 1: Access the survey online by clicking **HERE**.

Option 2: Complete the survey on paper by downloading a .pdf version available by clicking **HERE,** or by visiting either the UCS website ([www.ucsusa.org/2018survey](http://www.ucsusa.org/2018survey)) or the CSSM website ([www.cssm.iastate.edu/federal-](http://www.cssm.iastate.edu/federal-scientists-survey) [scientists-survey](http://www.cssm.iastate.edu/federal-scientists-survey)).

Option 3: Complete the survey via telephone by calling 877-578-8848 (toll-free) to schedule a time with Iowa State University’s CSSM project staff.

Completing the survey should take approximately 15-20 minutes of your time. If your work does not involve science, there is a place to indicate this near the beginning of the survey, and you will not be contacted again after submitting this response.

If you choose to opt out of the survey, please click **HERE** to remove your email address from future notifications. Your participation in this project is voluntary, and you may decline to answer any questions you choose. However, your participation is extremely important since a high response rate is essential to high quality data.

Because this survey is not part of your official duties, we recommend that you complete the survey outside of work hours and on your personal computer. The online survey is smartphone compatible but easier to complete

on a larger device.

For more than 13 years, UCS (a 501(c)3 nonprofit that works on a variety of science policy issues) has conducted surveys of scientists at federal agencies, including the Food and Drug Administration, the Fish and Wildlife Service, and the Environmental Protection Agency. To learn more about prior surveys conducted by UCS, please visit [www.ucsusa.org/surveys](http://www.ucsusa.org/surveys).

Thank you in advance for your important and highly valued contribution to this research. If you have questions about the survey topic/purpose, please email [ScientistSurvey@ucsusa.org](mailto:ScientistSurvey@ucsusa.org) or call 202-331-6942. For technical assistance, please email CSSM at [cssmqualtrics@iastate.edu.](mailto:cssmqualtrics@iastate.edu)

Sincerely,

Jacob Carter, Ph.D., Research Scientist, Union of Concerned Scientists

Gretchen Goldman, Ph.D., Research Director, Union of Concerned Scientists

Charise Johnson, M.S., Research Associate, Union of Concerned Scientists

Zhengyuan Zhu, Ph.D., Professor of Statistics and Director of Center for Survey Statistics and Methodology, Iowa State University

This study has been approved by the Iowa State University Institutional Review Board (IRB #18-017) to ensure confidentiality and data security.

To opt out of the survey, click **HERE**.
