# Supplementary material for: Perceived losses of scientific integrity under the Trump administration: A survey of federal scientists
Source: PLoS One. 2020 Apr 23;15(4):e0231929. doi: 10.1371/journal.pone.0231929 (PMC7179855; doi:10.1371/journal.pone.0231929)
Supplement: S3 Appendix — (DOCX) [file pone.0231929.s003.docx]

# APPENDIX C. 2018 Survey of Federal Scientists (Paper Version)

Thank you for your willingness to complete this anonymous survey of scientists affiliated with the federal agencies

listed below. Approximately 63,000 scientists have been invited to complete this survey. Please write legibly and respond thoughtfully and honestly to each question. Unless it is otherwise noted, circle one response for each item. Return your completed survey to the address provided after the final question. For more information about this survey, please visit [www.ucsusa.org/2018survey.](http://www.ucsusa.org/2018survey)

## Introduction.

**Note: Please enter the access code from your email:**

1. **What is your primary affiliation?**

1 = ARS USDA Agricultural Research Service 2 = BOEM Bureau of Ocean Management

3 = BSEE Bureau of Safety and Environmental Enforcement 4 = CDC Centers for Disease Control and Prevention

5 = EERE US Department of Energy Office of Energy Efficiency & Renewable Energy 6 = EPA US Environmental Protection Agency

7 = ERS USDA Economic Research Service 8 = FDA US Food and Drug Administration

9 = NASS USDA National Agricultural Statistics Service

10 = NIFA USDA National Institute of Food and Agriculture 11 = NOAA National Oceanic and Atmospheric Administration 12 = USFWS US Fish and Wildlife Service

13 = USGS US Geological Survey 14 = US Census Bureau

15 = US National Park Service

16 = NHTSA National Highway Traffic Safety Administration

17 = Other (Please explain: )

1. **What division do you work in within your agency?**

**3. Approximately what percentage of your job duties involves science?**

(Note: Scientific work may include, but is not limited to, basic research, laboratory testing, data collection, risk assessment, veterinary medicine, economic analysis, science policy and other topics.)

1 = None [IF NONE, PLEASE GO TO QUESTION 58, PAGE 14.]

2 = 1-25% scientific work

3 = 26-50% scientific work

4 = 51-75% scientific work

5 = 76-100% scientific work

Iowa State University Center for Survey Statistics & Methodology Union of Concerned Scientists

**Survey of Federal Scientists**

**2018**

## Agency Mission and Effectiveness.

Preamble: The following questions aim to understand federal scientists’ attitudes regarding the mission and effectiveness of your agency. Effectiveness is defined in this survey as the ability to meet intended or expected outcomes as per the agency’s mission.

1. **Compared to one year ago, the effectiveness of your division or office has:**

| Increased | Stayed the Same | Decreased | Don’t Know | Not Applicable | Prefer Not to Disclose |
| --- | --- | --- | --- | --- | --- |
| 1 | 2 | 3 | 4 | 5 | 6 |

1. **Over the past year, your personal job satisfaction at your agency has:**

| Increased | Stayed the Same | Decreased | Don’t Know | Not Applicable | Prefer Not to Disclose |
| --- | --- | --- | --- | --- | --- |
| 1 | 2 | 3 | 4 | 5 | 6 |

**6. How would you rate morale within your center/office/service?**

| Excellent | Good | Fair | Poor | Extremely Poor | Don’t Know | Prefer Not to Disclose |
| --- | --- | --- | --- | --- | --- | --- |
| 1 | 2 | 3 | 4 | 5 | 6 | 7 |

Please indicate the extent to which you agree or disagree with the following statements.

**7a. In the last year, I have noticed workforce reductions at my agency due to staff departures, retirements, and/or hiring freezes.**

| Strongly Disagree | Disagree | Do Not Agree or Disagree | Agree | Strongly Agree | Prefer Not to Disclose |
| --- | --- | --- | --- | --- | --- |
| 1 | 2 | 3 | 4 | 5 | 6 |

**7b.** [IF Q7a = Agree or Strongly Agree:]

**Such workforce reductions have made it more difficult for my agency to fulfill its science-based mission.**

| Strongly Disagree | Disagree | Do Not Agree or Disagree | Agree | Strongly Agree | Prefer Not to Disclose |
| --- | --- | --- | --- | --- | --- |
| 1 | 2 | 3 | 4 | 5 | 6 |

**8. Thinking about the past year, the mix of tasks I am asked to perform is relevant to my expertise and job description.**

| Strongly Disagree | Disagree | Do Not Agree or Disagree | Agree | Strongly Agree | Prefer Not to Disclose |
| --- | --- | --- | --- | --- | --- |
| 1 | 2 | 3 | 4 | 5 | 6 |

1. **Over the past year, I have noticed that resource allocations (e.g., funding, staff time) have been distributed away from programs and offices whose work is viewed as politically contentious.**

| Strongly Disagree | Disagree | Do Not Agree or Disagree | Agree | Strongly Agree | Prefer Not to Disclose |
| --- | --- | --- | --- | --- | --- |
| 1 | 2 | 3 | 4 | 5 | 6 |

1. **In your opinion, what are the greatest barriers to science-based decisions at your agency?**

*(Circle up to 3.)*

1 = Delay in leadership making a decision

2 = Absence of leadership with needed scientific expertise 3 = Uncertainty or disagreement with the science

4 = Influence of political appointees in your agency or department 5 = Influence of the White House

6 = Influence of Congress

7 = Influence of other agencies

8 = Influence of industry stakeholders

9 = Influence of non-governmental interests (such as advocacy groups) 10 = Inefficient decision-making process within the Agency

11 = Potential discrepancy with existing rules or regulations 12 = Uncertainty of Agency jurisdiction

13 = Complexity of the issue 14 = Limited staff capacity

15 = Other (Please describe: ) 16 = Prefer Not to Disclose

## Employee Working Environment.

Please indicate the extent to which you agree or disagree with the following statements.

1. **My direct supervisor consistently stands behind scientists who put forth scientifically defensible positions that may be politically contentious.**

| Strongly Disagree | Disagree | Do Not Agree or Disagree | Agree | Strongly Agree | Prefer Not to Disclose |
| --- | --- | --- | --- | --- | --- |
| 1 | 2 | 3 | 4 | 5 | 6 |

1. **I am provided adequate time and resources to keep up with advances in my profession, such as attending conferences and trainings, and participation in scientific or professional societies.**

| Strongly Disagree | Disagree | Do Not Agree or Disagree | Agree | Strongly Agree | Prefer Not to Disclose |
| --- | --- | --- | --- | --- | --- |
| 1 | 2 | 3 | 4 | 5 | 6 |

1. **The number of scientific conferences attended by agency scientists in the past year is similar to the number attended three years ago.**

| Strongly Disagree | Disagree | Do Not Agree or Disagree | Agree | Strongly Agree | Prefer Not to Disclose |
| --- | --- | --- | --- | --- | --- |
| 1 | 2 | 3 | 4 | 5 | 6 |

1. **Currently, I can openly express any concerns about the mission-driven work of my agency without fear of retaliation (i.e., inappropriate criticism or consequences).**

| Strongly Disagree | Disagree | Do Not Agree or Disagree | Agree | Strongly Agree | Prefer Not to Disclose |
| --- | --- | --- | --- | --- | --- |
| 1 | 2 | 3 | 4 | 5 | 6 |

1. **I have been asked or told to omit certain words in my scientific work products because they are politically contentious.**

| Strongly Disagree | Disagree | Do Not Agree or Disagree | Agree | Strongly Agree | Prefer Not to Disclose |
| --- | --- | --- | --- | --- | --- |
| 1 | 2 | 3 | 4 | 5 | 6 |

**16a. I have been asked or told to avoid work on specific scientific topics because they are politically contentious.**

| Strongly Disagree | Disagree | Do Not Agree or Disagree | Agree | Strongly Agree | Prefer Not to Disclose |
| --- | --- | --- | --- | --- | --- |
| 1 | 2 | 3 | 4 | 5 | 6 |

**16b.** [IF Q16a = Agree or Strongly Agree:]

**This has adversely impacted my effectiveness at my job at my agency.**

| Strongly Disagree | Disagree | Do Not Agree or Disagree | Agree | Strongly Agree | Prefer Not to Disclose |
| --- | --- | --- | --- | --- | --- |
| 1 | 2 | 3 | 4 | 5 | 6 |

1. **I have avoided working on certain scientific topics or using certain scientific terms because they are politically contentious, though I was not explicitly told to avoid them.**

| Strongly Disagree | Disagree | Do Not Agree or Disagree | Agree | Strongly Agree | Prefer Not to Disclose |
| --- | --- | --- | --- | --- | --- |
| 1 | 2 | 3 | 4 | 5 | 6 |

1. **Currently, I am allowed to publish work in peer-reviewed scientific journals regardless of the level of controversy of the topic.**

| Strongly Disagree | Disagree | Do Not Agree or Disagree | Agree | Strongly Agree | Prefer Not to Disclose |
| --- | --- | --- | --- | --- | --- |
| 1 | 2 | 3 | 4 | 5 | 6 |

1. **Currently, I am allowed to speak to the public and the news media (including at conferences and professional meetings) about my scientific research findings, regardless of the level of political contentiousness of the topic.**

| Strongly Disagree | Disagree | Do Not Agree or Disagree | Agree | Strongly Agree | Prefer Not to Disclose |
| --- | --- | --- | --- | --- | --- |
| 1 | 2 | 3 | 4 | 5 | 6 |

**Science.**

Please indicate how often you feel each of the following statements is true.

1. **My agency collects the scientific and monitoring information needed to effectively meet its mission.**

| Always | Frequently | Occasionally | Seldom | Never | Don’t Know | Prefer Not to Disclose |
| --- | --- | --- | --- | --- | --- | --- |
| 1 | 2 | 3 | 4 | 5 | 6 | 7 |

1. **My agency’s determinations and actions are consistent with the scientific findings contained in agency documents and reports.**

| Always | Frequently | Occasionally | Seldom | Never | Don’t Know | Prefer Not to Disclose |
| --- | --- | --- | --- | --- | --- | --- |
| 1 | 2 | 3 | 4 | 5 | 6 | 7 |

1. **Expert advice from scientific advisory committees is heeded and incorporated into agency decisions.**

| Always | Frequently | Occasionally | Seldom | Never | Don’t Know | Prefer Not to Disclose |
| --- | --- | --- | --- | --- | --- | --- |
| 1 | 2 | 3 | 4 | 5 | 6 | 7 |

Please indicate the extent to which you agree or disagree with the following statements.

1. **I feel that my scientific work and opinions consistently inform policy decisions.**

| Strongly Disagree | Disagree | Do Not Agree or Disagree | Agree | Strongly Agree | Prefer Not to Disclose |
| --- | --- | --- | --- | --- | --- |
| 1 | 2 | 3 | 4 | 5 | 6 |

1. **In the past year, I have been excluded from discussions or decisions related to my scientific work that I normally would expect to be a part of.**

| Strongly Disagree | Disagree | Do Not Agree or Disagree | Agree | Strongly Agree | Prefer Not to Disclose |
| --- | --- | --- | --- | --- | --- |
| 1 | 2 | 3 | 4 | 5 | 6 |

1. **I have been pressured or directed by supervisors or senior level officials to NOT attend professional meetings or give public talks on my scientific work.**

| Strongly Disagree | Disagree | Do Not Agree or Disagree | Agree | Strongly Agree | Prefer Not to Disclose |
| --- | --- | --- | --- | --- | --- |
| 1 | 2 | 3 | 4 | 5 | 6 |

1. **Advisory committees at my agency are constituted by individuals with appropriate expertise and who are able to provide independent scientific advice.**

| Strongly Disagree | Disagree | Do Not Agree or Disagree | Agree | Strongly Agree | Prefer Not to Disclose |
| --- | --- | --- | --- | --- | --- |
| 1 | 2 | 3 | 4 | 5 | 6 |

**27. In the last year, the expertise on scientific advisory committees at my agency has:**

| Significantly Improved | Somewhat Improved | No Change | Somewhat Deteriorated | Significantly Deteriorated | Don’t Know | Prefer Not to Disclose |
| --- | --- | --- | --- | --- | --- | --- |
| 1 | 2 | 3 | 4 | 5 | 6 | 7 |

## Outside Influence & Political Interference.

Please indicate the extent to which you agree or disagree with the following statements.

**28a. The level of consideration of political interests hinders the ability of my agency to make science-based decisions.**

| Strongly Disagree | Disagree | Do not agree or disagree | Agree | Strongly Agree | Prefer Not to Disclose |
| --- | --- | --- | --- | --- | --- |
| 1 | 2 | 3 | 4 | 5 | 6 |

**28b. The level of consideration of business interests hinders the ability of my agency to make science-based decisions.**

| Strongly Disagree | Disagree | Do not agree or disagree | Agree | Strongly Agree | Prefer Not to Disclose |
| --- | --- | --- | --- | --- | --- |
| 1 | 2 | 3 | 4 | 5 | 6 |

**28c. The level of consideration of non-governmental organization interests hinders the ability of my agency to make science-based decisions.**

| Strongly Disagree | Disagree | Do not agree or disagree | Agree | Strongly Agree | Prefer Not to Disclose |
| --- | --- | --- | --- | --- | --- |
| 1 | 2 | 3 | 4 | 5 | 6 |

**28d. The level of consideration of public opinion hinders the ability of my agency to make science-based decisions.**

| Strongly Disagree | Disagree | Do not agree or disagree | Agree | Strongly Agree | Prefer Not to Disclose |
| --- | --- | --- | --- | --- | --- |
| 1 | 2 | 3 | 4 | 5 | 6 |

**29. The presence of senior decision makers in my agency who come from regulated industry or those with financial interest in regulatory outcomes inappropriately influences the decisions made by the agency.**

| Strongly Disagree | Disagree | Do Not Agree or Disagree | Agree | Strongly Agree | Prefer Not to Disclose |
| --- | --- | --- | --- | --- | --- |
| 1 | 2 | 3 | 4 | 5 | 6 |

**Interaction with the Public and the Press.**

**30. Are you able to review, prior to publication, the final drafts of agency communications that are being published under your name and/or that substantially rely on your research?**

1 = Yes

2 = No

3 = Don’t Know

4 = Prefer Not to Disclose

**31a. Are you required to obtain agency pre-approval to communicate with journalists?**

1 = Yes

2 = No

3 = Don’t Know

4 = Prefer Not to Disclose

**31b**. [IF Q31a = Yes:] **Has this affected your ability to communicate your science externally?**

1 = Yes

2 = No

3 = Don’t Know

4 = Prefer Not to Disclose

**32. In the past year, how has your ability to communicate your scientific work to the public and to the media, including the use of social media, changed?**

| Significantly Improved | Somewhat Improved | No Change | Somewhat Deteriorated | Significantly Deteriorated | Don’t Know | Prefer Not to Disclose |
| --- | --- | --- | --- | --- | --- | --- |
| 1 | 2 | 3 | 4 | 5 | 6 | 7 |

## Agency Scientific Integrity and Whistleblower Policies.

Each agency included in this survey has a scientific integrity policy that is intended to safeguard the integrity of agency science by setting clear standards for employees and the use of scientific information, and institutes a process for resolving disputes. Please indicate the extent to which you agree or disagree with each of the following statements.

1. **My agency adheres to its scientific integrity policy.**

| Strongly Disagree | Disagree | Do Not Agree or Disagree | Agree | Strongly Agree | Don’t Know | Prefer Not to Disclose |
| --- | --- | --- | --- | --- | --- | --- |
| 1 | 2 | 3 | 4 | 5 | 6 | 7 |

1. **I have received adequate training regarding the contents and procedures in my agency’s scientific integrity policy.**

| Strongly Disagree | Disagree | Do Not Agree or Disagree | Agree | Strongly Agree | Prefer Not to Disclose |
| --- | --- | --- | --- | --- | --- |
| 1 | 2 | 3 | 4 | 5 | 6 |

Among other protections, federal whistleblower laws (including the Whistleblower Protection Act and the Whistleblower Protection Enhancement Act) protect federal scientists from retaliation who report information they reasonably believe displays:

- A violation of law, rule or regulation;
- Gross mismanagement;
- A gross waste of funds;
- Abuse of authority;
- A substantial and specific danger to public health and safety; or
- Censorship related to research, analysis or technical information that is, or will cause, any of the above forms of misconduct

To what extent do you disagree or agree with the following statement?

1. **I have been adequately trained on whistleblower rights and protections.**

| Strongly Disagree | Disagree | Do Not Agree or Disagree | Agree | Strongly Agree | Prefer Not to Disclose |
| --- | --- | --- | --- | --- | --- |
| 1 | 2 | 3 | 4 | 5 | 6 |

1. **If I were to obtain knowledge about a scientific integrity issue, I would most likely:**

(Examples of scientific integrity issues include political appointees changing data in a scientific report, scientists being restricted from talking to the media/public, suppression of scientific information that could result in public harm, undue influence of outside stakeholders on agency scientific decisions, etc.)

1 = Be willing to come forward. I trust that the agency would fairly assess and address the issue. 2 = Be willing to come forward, although I do not trust that the agency would fairly assess and

address the issue.

3 = Not feel comfortable coming forward. I do not trust that the agency would fairly assess and address the issue.

4 = Not feel comfortable coming forward, although I do trust that the agency would fairly assess and address the issue.

5 = Not feel comfortable coming forward because I would fear suffering retaliation for reporting the violation.

6 = Don’t Know

7 = Prefer Not to Disclose

***NOTE:***

***FDA SCIENTISTS: Skip to Q40a, next page. NHTSA SCIENTISTS: Skip to Q45, page 12.***

***CENSUS BUREAU SCIENTISTS: Skip to Q50, page 13.***

***ALL OTHERS CONTINUE BELOW.***

Please indicate the extent to which you agree or disagree with each of the following statements.

**37. I have been asked or told to omit the phrase “climate change” from my work.**

| Strongly Disagree | Disagree | Do Not Agree or Disagree | Agree | Strongly Agree | Prefer Not to Disclose |
| --- | --- | --- | --- | --- | --- |
| 1 | 2 | 3 | 4 | 5 | 6 |

**38a. I have been asked or told to avoid work on climate change.**

| Strongly Disagree | Disagree | Do Not Agree or Disagree | Agree | Strongly Agree | Prefer Not to Disclose |
| --- | --- | --- | --- | --- | --- |
| 1 | 2 | 3 | 4 | 5 | 6 |

**38b.** [IF Q38a = Agree or Strongly Agree:]

**This has adversely impacted my effectiveness at my job at my agency.**

| Strongly Disagree | Disagree | Do Not Agree or Disagree | Agree | Strongly Agree | Prefer Not to Disclose |
| --- | --- | --- | --- | --- | --- |
| 1 | 2 | 3 | 4 | 5 | 6 |

**39. I have avoided working on climate change or using the phrase “climate change,” though I was not explicitly told to avoid them.**

| Strongly Disagree | Disagree | Do Not Agree or Disagree | Agree | Strongly Agree | Prefer Not to Disclose |
| --- | --- | --- | --- | --- | --- |
| 1 | 2 | 3 | 4 | 5 | 6 |

20

**Agency Specific Questions: FOR FDA SCIENTISTS ONLY. (All others, go to next page.)**

Please indicate the extent to which you agree or disagree with each of the following statements.

**40a. Rules and regulations supported by scientific evidence that were final or near final have been delayed.**

| Strongly Disagree | Disagree | Do Not Agree or Disagree | Agree | Strongly Agree | Prefer Not to Disclose |
| --- | --- | --- | --- | --- | --- |
| 1 | 2 | 3 | 4 | 5 | 6 |

**40b.** [IF Q40a = Agree or Strongly Agree:] **Which rules and regulations have been delayed?**

*(Please explain below.)*

**41. I have felt pressure to lower or relax scientific, clinical reviews, or inspection standards.**

| Strongly Disagree | Disagree | Do Not Agree or Disagree | Agree | Strongly Agree | Prefer Not to Disclose |
| --- | --- | --- | --- | --- | --- |
| 1 | 2 | 3 | 4 | 5 | 6 |

**42a. Time pressures have undermined the scientific rigor of my work.**

| Strongly Disagree | Disagree | Do Not Agree or Disagree | Agree | Strongly Agree | Prefer Not to Disclose |
| --- | --- | --- | --- | --- | --- |
| 1 | 2 | 3 | 4 | 5 | 6 |

**42b.** [IF Q42a = Agree or Strongly Agree:]

**Is this related to deadlines and user fee requirements?**

1 = Yes

2 = No

3 = Don’t Know

4 = Prefer Not to Disclose

**Agency Specific Questions: FOR USDA (ARS, ERS, NIFA, NASS) SCIENTISTS ONLY.**

**(All others, go to next page.)**

Please indicate the extent to which you agree or disagree with each of the following statements.

**43a. My agency has received input or guidance on its scientific work or other information about cross-departmental scientific coordination from the USDA’s Office of the Chief Scientist (OCS) during the past year.**

| Strongly Disagree | Disagree | Do Not Agree or Disagree | Agree | Strongly Agree | Prefer Not to Disclose |
| --- | --- | --- | --- | --- | --- |
| 1 | 2 | 3 | 4 | 5 | 6 |

**43b.** [IF Q43a = Disagree or Strongly Disagree:]

**This lack of input/feedback about cross-departmental scientific coordination is a departure from previous OCS practice.**

| Strongly Disagree | Disagree | Do Not Agree or Disagree | Agree | Strongly Agree | Prefer Not to Disclose |
| --- | --- | --- | --- | --- | --- |
| 1 | 2 | 3 | 4 | 5 | 6 |

**44a. Over the past year, the OCS has convened the USDA Science Council about as regularly as in previous years.**

| Strongly Disagree | Disagree | Do Not Agree or Disagree | Agree | Strongly Agree | Prefer Not to Disclose |
| --- | --- | --- | --- | --- | --- |
| 1 | 2 | 3 | 4 | 5 | 6 |

**44b.** [IF Q44a = Disagree or Strongly Disagree:]

**This has hampered cross-departmental scientific coordination and collaboration and made it more difficult for my agency to fulfill its science-based mission.**

| Strongly Disagree | Disagree | Do Not Agree or Disagree | Agree | Strongly Agree | Prefer Not to Disclose |
| --- | --- | --- | --- | --- | --- |
| 1 | 2 | 3 | 4 | 5 | 6 |

**Agency Specific Questions: FOR BOEM, BSEE, & NHTSA SCIENTISTS ONLY.**

**(All others, go to next page.)**

Please indicate the extent to which you agree or disagree with each of the following statements.

1. **Current procedures for assessing the environmental impacts of my agency’s actions are adequate.**

| Strongly Disagree | Disagree | Do Not Agree or Disagree | Agree | Strongly Agree | Prefer Not to Disclose |
| --- | --- | --- | --- | --- | --- |
| 1 | 2 | 3 | 4 | 5 | 6 |

1. **Current procedures for assessing the climate change impacts of my agency’s actions are adequate.**

| Strongly Disagree | Disagree | Do Not Agree or Disagree | Agree | Strongly Agree | Prefer Not to Disclose |
| --- | --- | --- | --- | --- | --- |
| 1 | 2 | 3 | 4 | 5 | 6 |

**47a. In the past year, rules and regulations supported by scientific evidence that were final or near final have been delayed or overturned.**

| Strongly Disagree | Disagree | Do Not Agree or Disagree | Agree | Strongly Agree | Prefer Not to Disclose |
| --- | --- | --- | --- | --- | --- |
| 1 | 2 | 3 | 4 | 5 | 6 |

**47b.** [IF Q47a = Agree or Strongly Agree:] **Which rules and regulations have been delayed or overturned?**

*(Please explain below.)*

**48a. In the past year, I have felt pressure or was directed to lower or relax scientific or analytic standards.**

| Strongly Disagree | Disagree | Do Not Agree or Disagree | Agree | Strongly Agree | Prefer Not to Disclose |
| --- | --- | --- | --- | --- | --- |
| 1 | 2 | 3 | 4 | 5 | 6 |

**48b. In recent years, increasing time pressures have undermined the scientific rigor of my work.**

| Strongly Disagree | Disagree | Do Not Agree or Disagree | Agree | Strongly Agree | Prefer Not to Disclose |
| --- | --- | --- | --- | --- | --- |
| 1 | 2 | 3 | 4 | 5 | 6 |

**49.** [NHTSA scientists only:] **Current practices for assessing the impacts of my agency’s actions on consumers adequately consider scientific evidence.**

| Strongly Disagree | Disagree | Do Not Agree or Disagree | Agree | Strongly Agree | Prefer Not to Disclose |
| --- | --- | --- | --- | --- | --- |
| 1 | 2 | 3 | 4 | 5 | 6 |

**Demographic Background. (For all respondents.)**

| **50. What is your current grade level?**  1 = GS-11 or lower  2 = GS-12 and above  3 = Commissioned Corps 4 = Student  5 = Fellow  6 = Visiting Scientist 7 = Contractor  8 = Other *(Please specify:*  *)*  9 = Prefer Not to Disclose | **53. What is the highest level of education you have completed?**  1 = Bachelor’s Degree 2 = Master’s Degree 3 = PhD  4 = MD  5 = JD  6 = DVM  7 = Other *(Please specify:*  *)*  8 = Prefer Not to Disclose |
| --- | --- |
| **51. How long have you been working at your agency?**  1 = Less than 3 years  2 = 3-5 years  3 = 6-10 years  4 = 11-15 years  5 = More than 15 years  6 = Prefer Not to Disclose | **54. To which racial or ethnic group(s) do you most identify?** *(Circle all that apply.)*  1 = White, Caucasian  2 = Hispanic or Latino/a  3 = Black or African American  4 = Native American or American Indian 5 = Asian / Pacific Islander  6 = Other  7 = Prefer Not to Disclose |
| **52. Have you ever worked for regulated industry or a group representing them?**  1 = Yes  2 = No  3 = Prefer Not to Disclose | **55. What is your gender?**  1 = Male  2 = Female  3 = Nonbinary  4 = Prefer Not to Disclose |

## Open-Ended Questions.

Preamble: The following questions are open-ended to allow respondents to share further details regarding scientific integrity at their agency. For confidentiality purposes, please refrain from including any identifying information in your responses such as names of current career employees. You can also choose to contact UCS outside of this survey to share information about anything that inhibits the role of science in policymaking. (See [https://www.ucsusa.org](https://www.ucsusa.org/)/2018survey .)

**56a. How have actions taken or changes made by the current administration related to science (positive or negative) helped or harmed your agency’s mission?**

**56b. In particular, have you observed any potential impacts of policy actions on low-income communities, sensitive subpopulations and people of a minority race/ethnicity?**

**57. Please describe any specific problems related to scientific integrity or examples where scientific integrity was upheld, that deserve more public attention.**

(e.g. inappropriate interference by commercial, political, or nongovernmental interests, selective or incomplete use of data, significant edits that change the meaning of findings, new or unusual administrative policies that impair your work, requests to use data or methods that are not credible, etc.)

**58. Is there anything else you would like to share with us regarding scientific integrity at your agency?**
