# Supplementary material for: Perceived losses of scientific integrity under the Trump administration: A survey of federal scientists
Source: PLoS One. 2020 Apr 23;15(4):e0231929. doi: 10.1371/journal.pone.0231929 (PMC7179855; doi:10.1371/journal.pone.0231929)
Supplement: S4 Appendix — (PDF) [file pone.0231929.s004.pdf]

Table of Q3 by Agency									
Q3 Approximately what percentage of your job duties involves science?									
Frequency<br>Row Pct<br>Col Pct	CDC	EPA	FDA	NOAA	NPS	USFWS	USGS	USDA	Energy Agencies
2 = 1 - 25%	59<br>16.08<br>9.75	83<br>22.62<br>18.49	15<br>4.09<br>4.24	94<br>25.61<br>8.12	20<br>5.45<br>8.66	43<br>11.72<br>11.94	10<br>2.72<br>1.78	14<br><br>4.71	24<br><br>17.27
3 = 26 - 50%	61<br>13.03<br>10.08	60<br>12.82<br>13.36	30<br>6.41<br>8.47	134<br>28.63<br>11.57	55<br>11.75<br>23.81	68<br>14.53<br>18.89	25<br>5.34<br>4.46	14<br><br>4.71	19<br><br>13.67
4 = 51 - 75%	130<br>13.39<br>21.49	114<br>11.74<br>25.39	60<br>6.18<br>16.95	278<br>28.63<br>24.01	69<br>7.11<br>29.87	114<br>11.74<br>31.67	100<br>10.3<br>17.83	43<br><br>14.48	43<br><br>30.94
5 = 76 - 100%	355<br>14.76<br>58.68	192<br>7.98<br>42.76	249<br>10.35<br>70.34	652<br>27.11<br>56.3	87<br>3.62<br>37.66	135<br>5.61<br>37.5	426<br>17.71<br>75.94	226<br><br>76.09	53<br><br>38.13
Total	605	449	354	1158	231	360	561	297	139

Table of Q4 by Agency									
Q4 Compared to one year ago, the effectiveness of your division or office has:									
Frequency<br>Row Pct<br>Col Pct	CDC	EPA	FDA	NOAA	NPS	USFWS	USGS	USDA	Energy Agencies
1 = Increased	129<br>19.52<br>21.32	22<br>3.33<br>4.9	87<br>13.16<br>24.58	258<br>39.03<br>22.28	22<br>3.33<br>9.52	37<br>5.6<br>10.28	36<br>5.45<br>6.42	27<br><br>9.09	25<br><br>17.99
2 = Stayed the Same	228<br>14.19<br>37.69	112<br>6.97<br>24.94	170<br>10.58<br>48.02	541<br>33.67<br>46.72	81<br>5.04<br>35.06	97<br>6.04<br>26.94	186<br>11.57<br>33.16	113<br><br>38.05	42<br><br>30.22
3 = Decreased	175<br>10.77<br>28.93	284<br>17.48<br>63.25	58<br>3.57<br>16.38	276<br>16.98<br>23.83	124<br>7.63<br>53.68	213<br>13.11<br>59.17	287<br>17.66<br>51.16	146<br><br>49.16	61<br><br>43.88
4 = Don’t Know	51<br>20.56<br>8.43	22<br>8.87<br>4.9	27<br>10.89<br>7.63	66<br>26.61<br>5.7	3<br>1.21<br>1.3	13<br>5.24<br>3.61	46<br>18.55<br>8.2	9<br><br>3.03	9<br><br>6.47

**5 = Not Applicable**	14<br>35<br>2.31	2<br>5<br>0.45	10<br>25<br>2.82	10<br>25<br>0.86	0<br>0<br>0	0<br>0<br>0	3<br>7.5<br>0.53	0<br><br>0.00	0<br><br>0.00
**6 = Prefer Not to Disclose**	8<br>26.67<br>1.32	7<br>23.33<br>1.56	2<br>6.67<br>0.56	7<br>23.33<br>0.6	1<br>3.33<br>0.43	0<br>0<br>0	3<br>10<br>0.53	2<br><br>0.67	2<br><br>1.44
**Total**	605	449	354	1158	231	360	561	297	139

Table of Q5 by Agency

Q5 Over the past year, your personal job satisfaction at your agency has:

**Frequency**<br>**Row Pct**<br>**Col Pct**	**CDC**	**EPA**	**FDA**	**NOAA**	**NPS**	**USFWS**	**USGS**	**USDA**	**Energy Agencies**
**1 = Increased**	124<br>20.53<br>20.53	35<br>5.79<br>7.8	75<br>12.42<br>21.19	194<br>32.12<br>16.8	23<br>3.81<br>9.96	32<br>5.3<br>8.89	48<br>7.95<br>8.59	32<br><br>10.77	28<br><br>20.14
**2 = Stayed the Same**	227<br>14.32<br>37.58	113<br>7.13<br>25.17	165<br>10.41<br>46.61	522<br>32.93<br>45.19	65<br>4.1<br>28.14	114<br>7.19<br>31.67	210<br>13.25<br>37.57	101<br><br>34.01	33<br><br>23.74
**3 = Decreased**	227<br>11.76<br>37.58	292<br>15.12<br>65.03	102<br>5.28<br>28.81	419<br>21.7<br>36.28	140<br>7.25<br>60.61	210<br>10.88<br>58.33	299<br>15.48<br>53.49	158<br><br>53.20	74<br><br>53.24
**4 = Don’t Know**	4<br>28.57<br>0.66	0<br>0<br>0	2<br>14.29<br>0.56	3<br>21.43<br>0.26	2<br>14.29<br>0.87	2<br>14.29<br>0.56	0<br>0<br>0	0<br><br>0.00	0<br><br>0.00
**5 = Not Applicable**	16<br>34.78<br>2.65	2<br>4.35<br>0.45	6<br>13.04<br>1.69	14<br>30.43<br>1.21	0<br>0<br>0	1<br>2.17<br>0.28	2<br>4.35<br>0.36	4<br><br>1.35	3<br><br>2.16
**6 = Prefer Not to Disclose**	6<br>25<br>0.99	7<br>29.17<br>1.56	4<br>16.67<br>1.13	3<br>12.5<br>0.26	1<br>4.17<br>0.43	1<br>4.17<br>0.28	0<br>0<br>0	2<br><br>0.67	1<br><br>0.72
**Total**	604	449	354	1155	231	360	559	297	139

Table of Q6 by Agency

Q6 How would you rate morale within your center/office/service?

Frequency<br>Row Pct<br>Col Pct	CDC	EPA	FDA	NOAA	NPS	USFWS	USGS	USDA	Energy Agencies
**1 = Excellent**	45<br>20.55<br>7.48	13<br>5.94<br>2.9	41<br>18.72<br>11.65	64<br>29.22<br>5.55	11<br>5.02<br>4.76	6<br>2.74<br>1.67	18<br>8.22<br>3.22	10<br><br>3.39	5<br><br>3.65
**2 = Good**	206<br>16.8<br>34.22	45<br>3.67<br>10.04	157<br>12.81<br>44.6	380<br>31<br>32.96	35<br>2.85<br>15.15	88<br>7.18<br>24.44	150<br>12.23<br>26.83	84<br><br>28.47	43<br><br>31.39
**3 = Fair**	214<br>13.67<br>35.55	155<br>9.9<br>34.6	99<br>6.32<br>28.13	461<br>29.44<br>39.98	105<br>6.7<br>45.45	149<br>9.51<br>41.39	211<br>13.47<br>37.75	106<br><br>35.93	44<br><br>32.12
**4 = Poor**	86<br>10.29<br>14.29	152<br>18.18<br>33.93	40<br>4.78<br>11.36	199<br>23.8<br>17.26	57<br>6.82<br>24.68	86<br>10.29<br>23.89	129<br>15.43<br>23.08	64<br><br>21.69	29<br><br>21.17
**5 = Extremely Poor**	36<br>12.2<br>5.98	78<br>26.44<br>17.41	8<br>2.71<br>2.27	40<br>13.56<br>3.47	23<br>7.8<br>9.96	30<br>10.17<br>8.33	45<br>15.25<br>8.05	26<br><br>8.81	12<br><br>8.76
**6 = Don't Know**	7<br>24.14<br>1.16	3<br>10.34<br>0.67	3<br>10.34<br>0.85	5<br>17.24<br>0.43	0<br>0<br>0	1<br>3.45<br>0.28	6<br>20.69<br>1.07	1<br><br>0.34	1<br><br>0.73
**7 = Prefer Not to Disclose**	8<br>34.78<br>1.33	2<br>8.7<br>0.45	4<br>17.39<br>1.14	4<br>17.39<br>0.35	0<br>0<br>0	0<br>0<br>0	0<br>0<br>0	4<br><br>1.36	3<br><br>2.19
**Total**	602	448	352	1153	231	360	559	295	137

Table of Q7a by Agency									
**Q7a In the last year, I have noticed workforce reductions at my agency due to staff departures, retirements, and/or hiring freezes.**									
**Frequency<br>Row Pct<br>Col Pct**	**CDC**	**EPA**	**FDA**	**NOAA**	**NPS**	**USFWS**	**USGS**	**USDA**	**Energy Agencies**
**1 = Strongly Disagree**	20<br>13.16<br>3.38	16<br>10.53<br>3.58	20<br>13.16<br>5.68	52<br>34.21<br>4.52	7<br>4.61<br>3.03	5<br>3.29<br>1.39	8<br>5.26<br>1.44	10<br><br>3.40	7<br><br>5.11
**2 = Disagree**	45<br>14.24	10<br>3.16	73<br>23.1	116<br>36.71	6<br>1.9	8<br>2.53	24<br>7.59	4	15

	7.6	2.24	20.74	10.09	2.6	2.23	4.31	1.36	10.95
**3 = Do Not Agree or Disagree**	70	17	79	139	15	15	28	13	9
	17.59	4.27	19.85	34.92	3.77	3.77	7.04		
	11.82	3.8	22.44	12.09	6.49	4.18	5.03	4.42	6.57
**4 = Agree**	204	127	112	452	87	145	221	83	61
	13.52	8.42	7.42	29.95	5.77	9.61	14.65		
	34.46	28.41	31.82	39.3	37.66	40.39	39.68	28.23	44.53
**5 = Strongly Agree**	245	276	62	387	115	186	276	183	44
	13.76	15.51	3.48	21.74	6.46	10.45	15.51		
	41.39	61.74	17.61	33.65	49.78	51.81	49.55	62.24	32.12
**6 = Prefer Not to Disclose**	8	1	6	4	1	0	0	1	1
	38.1	4.76	28.57	19.05	4.76	0	0		
	1.35	0.22	1.7	0.35	0.43	0	0	0.34	0.73
**Total**	592	447	352	1150	231	359	557	294	137

Table of Q7b by Agency

Q7b Such workforce reductions have made it more difficult for my agency to fulfill its science-based mission.

**Frequency<br>Row Pct<br>Col Pct**	**CDC**	**EPA**	**FDA**	**NOAA**	**NPS**	**USFWS**	**USGS**	**USDA**	**Energy Agencies**
**1 = Strongly Disagree**	3	7	2	9	0	4	3	3	2
	9.09	21.21	6.06	27.27	0	12.12	9.09		
	0.67	1.74	1.15	1.07	0	1.21	0.6	1.13	1.92
**2 = Disagree**	9	12	13	20	3	3	14	3	7
	10.59	14.12	15.29	23.53	3.53	3.53	16.47		
	2.01	2.99	7.47	2.39	1.49	0.91	2.82	1.13	6.73
**3 = Do Not Agree or Disagree**	37	55	20	64	4	22	45	15	18
	13.03	19.37	7.04	22.54	1.41	7.75	15.85		
	8.26	13.68	11.49	7.64	1.98	6.65	9.07	5.64	17.31
**4 = Agree**	215	154	94	413	83	151	217	88	53
	14.55	10.42	6.36	27.94	5.62	10.22	14.68		
	47.99	38.31	54.02	49.28	41.09	45.62	43.75	33.08	50.96
**5 = Strongly Agree**	180	171	45	330	112	151	214	157	24
	12.94	12.29	3.24	23.72	8.05	10.86	15.38		
	40.18	42.54	25.86	39.38	55.45	45.62	43.15	59.02	23.08
**6 = Prefer Not to Disclose**	4	3	0	2	0	0	3	0	0
	30.77	23.08	0	15.38	0	0	23.08		

	CDC	EPA	FDA	NOAA	NPS	USFWS	USGS	USDA	Energy Agencies
	0.89	0.75	0	0.24	0	0	0.6	0.00	0.00
**Total**	448	402	174	838	202	331	496	266	104

Table of Q8 by Agency

Q8 Thinking about the past year, the mix of tasks I am asked to perform is relevant to my expertise and job description

Frequency<br>Row Pct<br>Col Pct	CDC	EPA	FDA	NOAA	NPS	USFWS	USGS	USDA	Energy Agencies
**1 = Strongly Disagree**	22<br>16.79<br>3.74	12<br>9.16<br>2.68	16<br>12.21<br>4.57	34<br>25.95<br>2.97	8<br>6.11<br>3.46	11<br>8.4<br>3.07	7<br>5.34<br>1.26	10<br><br>3.40	11<br><br>8.09
**2 = Disagree**	43<br>13.07<br>7.31	42<br>12.77<br>9.4	14<br>4.26<br>4	81<br>24.62<br>7.07	28<br>8.51<br>12.12	33<br>10.03<br>9.22	44<br>13.37<br>7.93	33<br><br>11.22	7<br><br>5.04
**3 = Do Not Agree or Disagree**	50<br>15.63<br>8.5	42<br>13.13<br>9.4	21<br>6.56<br>6	83<br>25.94<br>7.24	16<br>5<br>6.93	19<br>5.94<br>5.31	50<br>15.63<br>9.01	25<br><br>8.50	11<br><br>8.09
**4 = Agree**	293<br>14.5<br>49.83	226<br>11.19<br>50.56	162<br>8.02<br>46.29	556<br>27.52<br>48.52	121<br>5.99<br>52.38	190<br>9.41<br>53.07	260<br>12.87<br>46.85	134<br><br>45.58	63<br><br>46.32
**5 = Strongly Agree**	178<br>13.16<br>30.27	122<br>9.02<br>27.29	136<br>10.05<br>38.86	390<br>28.82<br>34.03	58<br>4.29<br>25.11	105<br>7.76<br>29.33	194<br>14.34<br>34.95	91<br><br>30.95	43<br><br>31.62
**6 = Prefer Not to Disclose**	2<br>22.22<br>0.34	3<br>33.33<br>0.67	1<br>11.11<br>0.29	2<br>22.22<br>0.17	0<br>0<br>0	0<br>0<br>0	0<br>0<br>0	1<br><br>0.34	1<br><br>0.74
**Total**	588	447	350	1146	231	358	555	294	136

Table of Q9 by Agency

Q9 Over the past year, I have noticed that resource allocations (e.g., funding, staff time) have been distributed away from programs and offices whose work is viewed as politically contentious.

Frequency<br>Row Pct<br>Col Pct	CDC	EPA	FDA	NOAA	NPS	USFWS	USGS	USDA	Energy Agencies
	19	14	28	87	1	11	23	20	10

**1 = Strongly Disagree**	8.8<br>3.26	6.48<br>3.15	12.96<br>8.07	40.28<br>7.62	0.46<br>0.43	5.09<br>3.07	10.65<br>4.15	6.83	7.41
**2 = Disagree**	74<br>10.93<br>12.69	23<br>3.4<br>5.17	86<br>12.7<br>24.78	278<br>41.06<br>24.34	21<br>3.1<br>9.13	32<br>4.73<br>8.94	64<br>9.45<br>11.55	50<br>17.06	24<br>17.78
**3 = Do Not Agree or Disagree**	205<br>14.46<br>35.16	114<br>8.04<br>25.62	154<br>10.86<br>44.38	443<br>31.24<br>38.79	79<br>5.57<br>34.35	118<br>8.32<br>32.96	131<br>9.24<br>23.65	110<br>37.54	34<br>25.19
**4 = Agree**	173<br>15.49<br>29.67	139<br>12.44<br>31.24	53<br>4.74<br>15.27	225<br>20.14<br>19.7	79<br>7.07<br>34.35	123<br>11.01<br>34.36	207<br>18.53<br>37.36	73<br>24.91	37<br>27.41
**5 = Strongly Agree**	91<br>14.77<br>15.61	145<br>23.54<br>32.58	13<br>2.11<br>3.75	83<br>13.47<br>7.27	49<br>7.95<br>21.3	67<br>10.88<br>18.72	121<br>19.64<br>21.84	32<br>10.92	23<br>17.04
**6 = Prefer Not to Disclose**	21<br>20.79<br>3.6	10<br>9.9<br>2.25	13<br>12.87<br>3.75	26<br>25.74<br>2.28	1<br>0.99<br>0.43	7<br>6.93<br>1.96	8<br>7.92<br>1.44	8<br>2.73	7<br>5.19
**Total**	583	445	347	1142	230	358	554	293	135

Table of Q10_1 by Agency

Q10_1 Delay in leadership making a decision

**Frequency<br>Row Pct<br>Col Pct**	**CDC**	**EPA**	**FDA**	**NOAA**	**NPS**	**USFWS**	**USGS**	**USDA**	**Energy Agencies**
**1 = Selected**	122<br>12.64<br>100	93<br>9.64<br>100	73<br>7.56<br>100	281<br>29.12<br>100	54<br>5.6<br>100	95<br>9.84<br>100	105<br>10.88<br>100	77<br>100.00	53<br>100.00
**Total**	122	93	73	281	54	95	105	77	53

Table of Q10_2 by Agency

Q10_2 Absence of leadership with needed scientific expertise

**Frequency<br>Row Pct<br>Col Pct**	**CDC**	**EPA**	**FDA**	**NOAA**	**NPS**	**USFWS**	**USGS**	**USDA**	**Energy Agencies**
**1 = Selected**	111	147	65	260	92	114	155	91	45

	10.27<br>100	13.6<br>100	6.01<br>100	24.05<br>100	8.51<br>100	10.55<br>100	14.34<br>100	100.00	100.00
**Total**	111	147	65	260	92	114	155	91	45

**Table of Q10_3 by Agency**<br>**Q10_3 Uncertainty or disagreement with the science**									
**Frequency**<br>**Row Pct**<br>**Col Pct**	**CDC**	**EPA**	**FDA**	**NOAA**	**NPS**	**USFWS**	**USGS**	**USDA**	**Energy Agencies**
**1 = Selected**	58<br>13.46<br>100	61<br>14.15<br>100	55<br>12.76<br>100	132<br>30.63<br>100	19<br>4.41<br>100	29<br>6.73<br>100	40<br>9.28<br>100	21<br>100.00	16<br>100.00
**Total**	58	61	55	132	19	29	40	21	16

**Table of Q10_4 by Agency**<br>**Q10_4 Influence of political appointees in your agency or department**									
**Frequency**<br>**Row Pct**<br>**Col Pct**	**CDC**	**EPA**	**FDA**	**NOAA**	**NPS**	**USFWS**	**USGS**	**USDA**	**Energy Agencies**
**1 = Selected**	129<br>10.78<br>100	270<br>22.56<br>100	35<br>2.92<br>100	192<br>16.04<br>100	105<br>8.77<br>100	166<br>13.87<br>100	185<br>15.46<br>100	74<br>100.00	41<br>100.00
**Total**	129	270	35	192	105	166	185	74	41

**Table of Q10_5 by Agency**<br>**Q10_5 Influence of the White House**									
**Frequency**<br>**Row Pct**<br>**Col Pct**	**CDC**	**EPA**	**FDA**	**NOAA**	**NPS**	**USFWS**	**USGS**	**USDA**	**Energy Agencies**
**1 = Selected**	190<br>19.53<br>100	122<br>12.54<br>100	42<br>4.32<br>100	217<br>22.3<br>100	54<br>5.55<br>100	82<br>8.43<br>100	162<br>16.65<br>100	62<br>100.00	29<br>100.00
**Total**	190	122	42	217	54	82	162	62	29

Table of Q10_6 by Agency									
Q10_6 Influence of Congress									
Frequency<br>Row Pct<br>Col Pct	CDC	EPA	FDA	NOAA	NPS	USFWS	USGS	USDA	Energy Agencies
1 = Selected	158<br>24.09<br>100	34<br>5.18<br>100	47<br>7.16<br>100	172<br>26.22<br>100	32<br>4.88<br>100	39<br>5.95<br>100	110<br>16.77<br>100	40<br><br>100.00	8<br><br>100.00
Total	158	34	47	172	32	39	110	40	8

Table of Q10_7 by Agency									
Q10_7 Influence of other agencies									
Frequency<br>Row Pct<br>Col Pct	CDC	EPA	FDA	NOAA	NPS	USFWS	USGS	USDA	Energy Agencies
1 = Selected	33<br>21.57<br>100	10<br>6.54<br>100	4<br>2.61<br>100	46<br>30.07<br>100	5<br>3.27<br>100	15<br>9.8<br>100	14<br>9.15<br>100	7<br><br>100.00	9<br><br>100.00
Total	33	10	4	46	5	15	14	7	9

Table of Q10_8 by Agency									
Q10_8 Influence of industry stakeholders									
Frequency<br>Row Pct<br>Col Pct	CDC	EPA	FDA	NOAA	NPS	USFWS	USGS	USDA	Energy Agencies
1 = Selected	51<br>7.82<br>100	152<br>23.31<br>100	87<br>13.34<br>100	178<br>27.3<br>100	18<br>2.76<br>100	77<br>11.81<br>100	42<br>6.44<br>100	21<br><br>100.00	20<br><br>100.00
Total	51	152	87	178	18	77	42	21	20

Table of Q10_9 by Agency

Q10_9 Influence of non-governmental interests (such as advocacy groups)									
Frequency<br>Row Pct<br>Col Pct	CDC	EPA	FDA	NOAA	NPS	USFWS	USGS	USDA	Energy Agencies
1 = Selected	56<br>16.09<br>100	28<br>8.05<br>100	47<br>13.51<br>100	104<br>29.89<br>100	17<br>4.89<br>100	31<br>8.91<br>100	22<br>6.32<br>100	17<br><br>100.00	16<br><br>100.00
Total	56	28	47	104	17	31	22	17	16

Table of Q10_10 by Agency									
Q10_10 Inefficient decision-making process within the Agency									
Frequency<br>Row Pct<br>Col Pct	CDC	EPA	FDA	NOAA	NPS	USFWS	USGS	USDA	Energy Agencies
1 = Selected	100<br>11.16<br>100	64<br>7.14<br>100	73<br>8.15<br>100	299<br>33.37<br>100	51<br>5.69<br>100	78<br>8.71<br>100	106<br>11.83<br>100	80<br><br>100.00	31<br><br>100.00
Total	100	64	73	299	51	78	106	80	31

Table of Q10_11 by Agency									
Q10_11 Potential discrepancy with existing rules or regulations									
Frequency<br>Row Pct<br>Col Pct	CDC	EPA	FDA	NOAA	NPS	USFWS	USGS	USDA	Energy Agencies
1 = Selected	26<br>11.82<br>100	27<br>12.27<br>100	37<br>16.82<br>100	45<br>20.45<br>100	14<br>6.36<br>100	19<br>8.64<br>100	16<br>7.27<br>100	17<br><br>100.00	15<br><br>100.00
Total	26	27	37	45	14	19	16	17	15

Table of Q10_12 by Agency									
Q10_12 Uncertainty of Agency jurisdiction									
Frequency	CDC	EPA	FDA	NOAA	NPS	USFWS	USGS	USDA	Energy Agencies

Row Pct<br>Col Pct									
1 = Selected	14<br>16.28<br>100	16<br>18.6<br>100	7<br>8.14<br>100	16<br>18.6<br>100	4<br>4.65<br>100	4<br>4.65<br>100	11<br>12.79<br>100	4<br><br>100.00	6<br><br>100.00
**Total**	14	16	7	16	4	4	11	4	6

**Table of Q10_13 by Agency**									
**Q10_13 Complexity of the issue**									
**Frequency<br>Row Pct<br>Col Pct**	**CDC**	**EPA**	**FDA**	**NOAA**	**NPS**	**USFWS**	**USGS**	**USDA**	**Energy Agencies**
**1 = Selected**	112<br>13.98<br>100	56<br>6.99<br>100	113<br>14.11<br>100	280<br>34.96<br>100	33<br>4.12<br>100	48<br>5.99<br>100	73<br>9.11<br>100	48<br><br>100.00	26<br><br>100.00
**Total**	112	56	113	280	33	48	73	48	26

**Table of Q10_14 by Agency**									
**Q10_14 Limited staff capacity**									
**Frequency<br>Row Pct<br>Col Pct**	**CDC**	**EPA**	**FDA**	**NOAA**	**NPS**	**USFWS**	**USGS**	**USDA**	**Energy Agencies**
**1 = Selected**	150<br>9.99<br>100	98<br>6.52<br>100	90<br>5.99<br>100	521<br>34.69<br>100	103<br>6.86<br>100	154<br>10.25<br>100	200<br>13.32<br>100	136<br><br>100.00	26<br><br>100.00
**Total**	150	98	90	521	103	154	200	136	26

**Table of Q10_15 by Agency**									
**Q10_15 Other**									
**Frequency<br>Row Pct<br>Col Pct**	**CDC**	**EPA**	**FDA**	**NOAA**	**NPS**	**USFWS**	**USGS**	**USDA**	**Energy Agencies**
**1 = Selected**	60	21	25	92	17	23	82	32	14

	CDC	EPA	FDA	NOAA	NPS	USFWS	USGS	USDA	Energy Agencies
	15.96<br>100	5.59<br>100	6.65<br>100	24.47<br>100	4.52<br>100	6.12<br>100	21.81<br>100	100.00	100.00
**Total**	60	21	25	92	17	23	82	32	14

**Table of Q10_16 by Agency**									
**Q10_16 Prefer Not to Disclose**									
**Frequency**<br>**Row Pct**<br>**Col Pct**	**CDC**	**EPA**	**FDA**	**NOAA**	**NPS**	**USFWS**	**USGS**	**USDA**	**Energy Agencies**
**1 = Selected**	34<br>24.64<br>100	8<br>5.8<br>100	21<br>15.22<br>100	36<br>26.09<br>100	5<br>3.62<br>100	1<br>0.72<br>100	16<br>11.59<br>100	13<br><br>100.00	3<br><br>100.00
**Total**	34	8	21	36	5	1	16	13	3

**Table of Q11 by Agency**									
**Q11 My direct supervisor consistently stands behind scientists who put forth scientifically defensible positions that may be politically contentious.**									
**Frequency**<br>**Row Pct**<br>**Col Pct**	**CDC**	**EPA**	**FDA**	**NOAA**	**NPS**	**USFWS**	**USGS**	**USDA**	**Energy Agencies**
**1 = Strongly Disagree**	21<br>11.54<br>3.7	21<br>11.54<br>4.78	11<br>6.04<br>3.24	53<br>29.12<br>4.67	12<br>6.59<br>5.33	21<br>11.54<br>5.95	17<br>9.34<br>3.1	14<br><br>4.78	9<br><br>6.62
**2 = Disagree**	39<br>15.23<br>6.87	33<br>12.89<br>7.52	31<br>12.11<br>9.14	66<br>25.78<br>5.81	16<br>6.25<br>7.11	23<br>8.98<br>6.52	16<br>6.25<br>2.91	18<br><br>6.14	10<br><br>7.35
**3 = Do Not Agree or Disagree**	129<br>15.69<br>22.71	90<br>10.95<br>20.5	63<br>7.66<br>18.58	233<br>28.35<br>20.53	33<br>4.01<br>14.67	66<br>8.03<br>18.7	86<br>10.46<br>15.66	71<br><br>24.23	29<br><br>21.32
**4 = Agree**	180<br>13.42<br>31.69	151<br>11.26<br>34.4	102<br>7.61<br>30.09	377<br>28.11<br>33.22	80<br>5.97<br>35.56	122<br>9.1<br>34.56	187<br>13.94<br>34.06	92<br><br>31.40	43<br><br>31.62
**5 = Strongly Agree**	183<br>12.84<br>32.22	134<br>9.4<br>30.52	120<br>8.42<br>35.4	388<br>27.23<br>34.19	82<br>5.75<br>36.44	119<br>8.35<br>33.71	240<br>16.84<br>43.72	93<br><br>31.74	44<br><br>32.35
	16	10	12	18	2	2	3	5	1

**6 = Prefer Not to Disclose**	23.19<br>2.82	14.49<br>2.28	17.39<br>3.54	26.09<br>1.59	2.9<br>0.89	2.9<br>0.57	4.35<br>0.55	1.71	0.74
**Total**	568	439	339	1135	225	353	549	4095	136

Table of Q12 by Agency

Q12 I am provided adequate time and resources to keep up with advances in my profession, such as attending conferences and trainings, and participation in scientific or professional societies.

Frequency<br>Row Pct<br>Col Pct	CDC	EPA	FDA	NOAA	NPS	USFWS	USGS	USDA	Energy Agencies
**1 = Strongly Disagree**	78<br>13.15<br>13.76	64<br>10.79<br>14.51	30<br>5.06<br>8.9	157<br>26.48<br>13.81	69<br>11.64<br>30.67	67<br>11.3<br>19.03	85<br>14.33<br>15.45	23<br>7.85	16<br>11.76
**2 = Disagree**	143<br>11.67<br>25.22	124<br>10.12<br>28.12	90<br>7.35<br>26.71	341<br>27.84<br>29.99	84<br>6.86<br>37.33	137<br>11.18<br>38.92	192<br>15.67<br>34.91	78<br>26.62	26<br>19.12
**3 = Do Not Agree or Disagree**	76<br>13.87<br>13.4	86<br>15.69<br>19.5	42<br>7.66<br>12.46	153<br>27.92<br>13.46	19<br>3.47<br>8.44	43<br>7.85<br>12.22	69<br>12.59<br>12.55	33<br>11.26	22<br>16.18
**4 = Agree**	200<br>15.86<br>35.27	116<br>9.2<br>26.3	118<br>9.36<br>35.01	350<br>27.76<br>30.78	45<br>3.57<br>20	80<br>6.34<br>22.73	161<br>12.77<br>29.27	111<br>37.88	54<br>39.71
**5 = Strongly Agree**	65<br>14.44<br>11.46	48<br>10.67<br>10.88	52<br>11.56<br>15.43	134<br>29.78<br>11.79	8<br>1.78<br>3.56	25<br>5.56<br>7.1	40<br>8.89<br>7.27	48<br>16.38	18<br>13.24
**6 = Prefer Not to Disclose**	5<br>26.32<br>0.88	3<br>15.79<br>0.68	5<br>26.32<br>1.48	2<br>10.53<br>0.18	0<br>0<br>0	0<br>0<br>0	3<br>15.79<br>0.55	0<br>0.00	0<br>0.00
**Total**	567	441	337	1137	225	352	550	293	136

Table of Q13 by Agency

Q13 The number of scientific conferences attended by agency scientists in the past year is similar to the number attended three years ago.

Frequency<br>Row Pct<br>Col Pct	CDC	EPA	FDA	NOAA	NPS	USFWS	USGS	USDA	Energy Agencies

**1 = Strongly Disagree**	89<br>12.36<br>15.78	74<br>10.28<br>16.89	29<br>4.03<br>8.58	138<br>19.17<br>12.19	48<br>6.67<br>21.33	102<br>14.17<br>28.98	195<br>27.08<br>35.39	18<br><br>6.14	21<br><br>15.56
**2 = Disagree**	178<br>12.96<br>31.56	155<br>11.29<br>35.39	81<br>5.9<br>23.96	364<br>26.51<br>32.16	97<br>7.06<br>43.11	131<br>9.54<br>37.22	226<br>16.46<br>41.02	84<br><br>28.67	42<br><br>31.11
**3 = Do Not Agree or Disagree**	179<br>17.79<br>31.74	126<br>12.52<br>28.77	100<br>9.94<br>29.59	276<br>27.44<br>24.38	49<br>4.87<br>21.78	80<br>7.95<br>22.73	77<br>7.65<br>13.97	72<br><br>24.57	33<br><br>24.44
**4 = Agree**	89<br>11.54<br>15.78	59<br>7.65<br>13.47	93<br>12.06<br>27.51	292<br>37.87<br>25.8	26<br>3.37<br>11.56	31<br>4.02<br>8.81	38<br>4.93<br>6.9	93<br><br>31.74	28<br><br>20.74
**5 = Strongly Agree**	12<br>8.76<br>2.13	14<br>10.22<br>3.2	20<br>14.6<br>5.92	41<br>29.93<br>3.62	2<br>1.46<br>0.89	7<br>5.11<br>1.99	13<br>9.49<br>2.36	20<br><br>6.83	7<br><br>5.19
**6 = Prefer Not to Disclose**	17<br>21.52<br>3.01	10<br>12.66<br>2.28	15<br>18.99<br>4.44	21<br>26.58<br>1.86	3<br>3.8<br>1.33	1<br>1.27<br>0.28	2<br>2.53<br>0.36	6<br><br>2.05	4<br><br>2.96
**Total**	564	438	338	1132	225	352	551	293	135

**Table of Q14 by Agency**									
**Q14 Currently, I can openly express any concerns about the mission-driven work of my agency without fear of retaliation (i.e., inappropriate criticism or consequences).**									
**Frequency**<br>**Row Pct**<br>**Col Pct**	**CDC**	**EPA**	**FDA**	**NOAA**	**NPS**	**USFWS**	**USGS**	**USDA**	**Energy Agencies**
**1 = Strongly Disagree**	49<br>13.88<br>8.75	77<br>21.81<br>17.54	27<br>7.65<br>8.01	70<br>19.83<br>6.17	16<br>4.53<br>7.11	44<br>12.46<br>12.57	34<br>9.63<br>6.18	23<br><br>7.93	15<br><br>11.36
**2 = Disagree**	123<br>15.34<br>21.96	114<br>14.21<br>25.97	48<br>5.99<br>14.24	187<br>23.32<br>16.49	61<br>7.61<br>27.11	83<br>10.35<br>23.71	113<br>14.09<br>20.55	46<br><br>15.86	27<br><br>20.45
**3 = Do Not Agree or Disagree**	109<br>12.59<br>19.46	97<br>11.2<br>22.1	78<br>9.01<br>23.15	233<br>26.91<br>20.55	52<br>6<br>23.11	67<br>7.74<br>19.14	125<br>14.43<br>22.73	68<br><br>23.45	22<br><br>16.67
**4 = Agree**	213<br>13.68<br>38.04	110<br>7.06<br>25.06	130<br>8.35<br>38.58	499<br>32.05<br>44	82<br>5.27<br>36.44	123<br>7.9<br>35.14	213<br>13.68<br>38.73	107<br><br>36.90	44<br><br>33.33

**5 = Strongly Agree**	51<br>11.94<br>9.11	30<br>7.03<br>6.83	46<br>10.77<br>13.65	135<br>31.62<br>11.9	13<br>3.04<br>5.78	27<br>6.32<br>7.71	56<br>13.11<br>10.18	39<br><br>13.45	19<br><br>14.39
**6 = Prefer Not to Disclose**	15<br>20.83<br>2.68	11<br>15.28<br>2.51	8<br>11.11<br>2.37	10<br>13.89<br>0.88	1<br>1.39<br>0.44	6<br>8.33<br>1.71	9<br>12.5<br>1.64	7<br><br>2.41	5<br><br>3.79
**Total**	560	439	337	1134	225	350	550	4077	132

Table of Q15 by Agency

Q15 I have been asked or told to omit certain words in my scientific work products because they are politically contentious.

**Frequency**<br>**Row Pct**<br>**Col Pct**	**CDC**	**EPA**	**FDA**	**NOAA**	**NPS**	**USFWS**	**USGS**	**USDA**	**Energy Agencies**
**1 = Strongly Disagree**	80<br>11.49<br>14.29	42<br>6.03<br>9.59	80<br>11.49<br>23.81	246<br>35.34<br>21.75	14<br>2.01<br>6.22	50<br>7.18<br>14.29	81<br>11.64<br>14.78	69<br><br>23.71	17<br><br>12.78
**2 = Disagree**	194<br>13.3<br>34.64	124<br>8.5<br>28.31	133<br>9.12<br>39.58	454<br>31.12<br>40.14	63<br>4.32<br>28	121<br>8.29<br>34.57	206<br>14.12<br>37.59	95<br><br>32.65	38<br><br>28.57
**3 = Do Not Agree or Disagree**	98<br>12.45<br>17.5	98<br>12.45<br>22.37	65<br>8.26<br>19.35	230<br>29.22<br>20.34	35<br>4.45<br>15.56	71<br>9.02<br>20.29	106<br>13.47<br>19.34	56<br><br>19.24	24<br><br>18.05
**4 = Agree**	109<br>15.08<br>19.46	104<br>14.38<br>23.74	38<br>5.26<br>11.31	152<br>21.02<br>13.44	72<br>9.96<br>32	69<br>9.54<br>19.71	103<br>14.25<br>18.8	39<br><br>13.40	22<br><br>16.54
**5 = Strongly Agree**	59<br>18.73<br>10.54	56<br>17.78<br>12.79	9<br>2.86<br>2.68	33<br>10.48<br>2.92	39<br>12.38<br>17.33	32<br>10.16<br>9.14	39<br>12.38<br>7.12	28<br><br>9.62	28<br><br>21.05
**6 = Prefer Not to Disclose**	20<br>21.98<br>3.57	14<br>15.38<br>3.2	11<br>12.09<br>3.27	16<br>17.58<br>1.41	2<br>2.2<br>0.89	7<br>7.69<br>2	13<br>14.29<br>2.37	4<br><br>1.37	4<br><br>3.01
**Total**	560	438	336	1131	225	350	548	4071	291

Table of Q16a by Agency

Q16a I have been asked or told to avoid work on specific scientific topics because they are politically contentious.

Frequency<br>Row Pct<br>Col Pct	CDC	EPA	FDA	NOAA	NPS	USFWS	USGS	USDA	Energy Agencies
1 = Strongly Disagree	86<br>12.25<br>15.41	36<br>5.13<br>8.24	81<br>11.54<br>24.32	274<br>39.03<br>24.25	17<br>2.42<br>7.56	41<br>5.84<br>11.75	69<br>9.83<br>12.59	65<br><br>22.41	19<br><br>14.39
2 = Disagree	221<br>13.42<br>39.61	131<br>7.95<br>29.98	148<br>8.99<br>44.44	501<br>30.42<br>44.34	93<br>5.65<br>41.33	140<br>8.5<br>40.11	236<br>14.33<br>43.07	103<br><br>35.52	40<br><br>30.30
3 = Do Not Agree or Disagree	98<br>11.61<br>17.56	111<br>13.15<br>25.4	63<br>7.46<br>18.92	224<br>26.54<br>19.82	55<br>6.52<br>24.44	79<br>9.36<br>22.64	117<br>13.86<br>21.35	66<br><br>22.76	25<br><br>18.94
4 = Agree	88<br>15.25<br>15.77	93<br>16.12<br>21.28	29<br>5.03<br>8.71	95<br>16.46<br>8.41	41<br>7.11<br>18.22	67<br>11.61<br>19.2	95<br>16.46<br>17.34	33<br><br>11.38	24<br><br>18.18
5 = Strongly Agree	48<br>21.72<br>8.6	53<br>23.98<br>12.13	6<br>2.71<br>1.8	24<br>10.86<br>2.12	17<br>7.69<br>7.56	17<br>7.69<br>4.87	24<br>10.86<br>4.38	17<br><br>5.86	20<br><br>15.15
6 = Prefer Not to Disclose	17<br>23.94<br>3.05	13<br>18.31<br>2.97	6<br>8.45<br>1.8	12<br>16.9<br>1.06	2<br>2.82<br>0.89	5<br>7.04<br>1.43	7<br>9.86<br>1.28	6<br><br>2.07	4<br><br>3.03
Total	558	437	333	1130	225	349	548	290	132

Table of Q16b by Agency									
Q16b This has adversely impacted my effectiveness at my job within my agency.									
Frequency<br>Row Pct<br>Col Pct	CDC	EPA	FDA	NOAA	NPS	USFWS	USGS	USDA	Energy Agencies
1 = Strongly Disagree	5<br>62.5<br>3.68	0<br>0<br>0	0<br>0<br>0	1<br>12.5<br>0.84	0<br>0<br>0	0<br>0<br>0	0<br>0<br>0	0<br><br>0.00	0<br><br>0.00
2 = Disagree	15<br>21.43<br>11.03	5<br>7.14<br>3.42	5<br>7.14<br>14.29	10<br>14.29<br>8.4	3<br>4.29<br>5.17	3<br>4.29<br>3.57	17<br>24.29<br>14.41	6<br><br>12.24	3<br><br>6.98
3 = Do Not Agree or Disagree	35<br>19.89<br>25.74	24<br>13.64<br>16.44	9<br>5.11<br>25.71	22<br>12.5<br>18.49	16<br>9.09<br>27.59	14<br>7.95<br>16.67	32<br>18.18<br>27.12	12<br><br>24.49	7<br><br>16.28

**4 = Agree**	51<br>13.82<br>37.5	79<br>21.41<br>54.11	14<br>3.79<br>40	63<br>17.07<br>52.94	26<br>7.05<br>44.83	46<br>12.47<br>54.76	49<br>13.28<br>41.53	20<br><br>40.82	19<br><br>44.19
**5 = Strongly Agree**	27<br>16.07<br>19.85	38<br>22.62<br>26.03	7<br>4.17<br>20	22<br>13.1<br>18.49	13<br>7.74<br>22.41	21<br>12.5<br>25	20<br>11.9<br>16.95	11<br><br>22.45	13<br><br>30.23
**6 = Prefer Not to Disclose**	3<br>60<br>2.21	0<br>0<br>0	0<br>0<br>0	1<br>20<br>0.84	0<br>0<br>0	0<br>0<br>0	0<br>0<br>0	0<br><br>0.00	1<br><br>2.33
**Total**	136	146	35	119	58	84	118	49	43

Table of Q17 by Agency

Q17 I have avoided working on certain scientific topics or using certain scientific terms because they are politically contentious, though I was not explicitly told to avoid them.

**Frequency<br>Row Pct<br>Col Pct**	**CDC**	**EPA**	**FDA**	**NOAA**	**NPS**	**USFWS**	**USGS**	**USDA**	**Energy Agencies**
**1 = Strongly Disagree**	96<br>13.69<br>17.24	44<br>6.28<br>10.09	82<br>11.7<br>24.7	238<br>33.95<br>21.12	23<br>3.28<br>10.22	58<br>8.27<br>16.71	68<br>9.7<br>12.41	54<br><br>18.69	26<br><br>19.85
**2 = Disagree**	198<br>13.73<br>35.55	133<br>9.22<br>30.5	125<br>8.67<br>37.65	428<br>29.68<br>37.98	86<br>5.96<br>38.22	127<br>8.81<br>36.6	187<br>12.97<br>34.12	92<br><br>31.83	37<br><br>28.24
**3 = Do Not Agree or Disagree**	115<br>14.11<br>20.65	84<br>10.31<br>19.27	75<br>9.2<br>22.59	224<br>27.48<br>19.88	49<br>6.01<br>21.78	69<br>8.47<br>19.88	103<br>12.64<br>18.8	57<br><br>19.72	22<br><br>16.79
**4 = Agree**	112<br>13.13<br>20.11	135<br>15.83<br>30.96	33<br>3.87<br>9.94	201<br>23.56<br>17.83	57<br>6.68<br>25.33	69<br>8.09<br>19.88	151<br>17.7<br>27.55	61<br><br>21.11	29<br><br>22.14
**5 = Strongly Agree**	25<br>13.37<br>4.49	31<br>16.58<br>7.11	8<br>4.28<br>2.41	26<br>13.9<br>2.31	10<br>5.35<br>4.44	19<br>10.16<br>5.48	36<br>19.25<br>6.57	22<br><br>7.61	13<br><br>9.92
**6 = Prefer Not to Disclose**	11<br>20<br>1.97	9<br>16.36<br>2.06	9<br>16.36<br>2.71	10<br>18.18<br>0.89	0<br>0<br>0	5<br>9.09<br>1.44	3<br>5.45<br>0.55	3<br><br>1.04	4<br><br>3.05
**Total**	557	436	332	1127	225	347	548	289	131

Table of Q18 by Agency

Q18 Currently, I am allowed to publish work in peer-reviewed scientific journals regardless of the level of controversy of the topic.									
**Frequency<br>Row Pct<br>Col Pct**	**CDC**	**EPA**	**FDA**	**NOAA**	**NPS**	**USFWS**	**USGS**	**USDA**	**Energy Agencies**
**1 = Strongly Disagree**	38<br>26.95<br>6.82	31<br>21.99<br>7.14	14<br>9.93<br>4.22	15<br>10.64<br>1.34	10<br>7.09<br>4.44	11<br>7.8<br>3.2	5<br>3.55<br>0.91	9<br><br>3.11	10<br><br>7.75
**2 = Disagree**	92<br>21.55<br>16.52	61<br>14.29<br>14.06	53<br>12.41<br>15.96	57<br>13.35<br>5.09	22<br>5.15<br>9.78	25<br>5.85<br>7.27	52<br>12.18<br>9.51	38<br><br>13.15	15<br><br>11.63
**3 = Do Not Agree or Disagree**	217<br>12.29<br>38.96	238<br>13.48<br>54.84	147<br>8.33<br>44.28	481<br>27.25<br>42.95	118<br>6.69<br>52.44	197<br>11.16<br>57.27	171<br>9.69<br>31.26	94<br><br>32.53	69<br><br>53.49
**4 = Agree**	151<br>11.98<br>27.11	64<br>5.08<br>14.75	86<br>6.83<br>25.9	423<br>33.57<br>37.77	62<br>4.92<br>27.56	80<br>6.35<br>23.26	252<br>20<br>46.07	109<br><br>37.72	21<br><br>16.28
**5 = Strongly Agree**	38<br>11.31<br>6.82	15<br>4.46<br>3.46	17<br>5.06<br>5.12	123<br>36.61<br>10.98	11<br>3.27<br>4.89	21<br>6.25<br>6.1	64<br>19.05<br>11.7	35<br><br>12.11	8<br><br>6.20
**6 = Prefer Not to Disclose**	21<br>19.27<br>3.77	25<br>22.94<br>5.76	15<br>13.76<br>4.52	21<br>19.27<br>1.88	2<br>1.83<br>0.89	10<br>9.17<br>2.91	3<br>2.75<br>0.55	4<br><br>1.38	6<br><br>4.65
**Total**	557	434	332	1120	225	344	547	289	129

Table of Q19 by Agency									
Q19 Currently, I am allowed to speak to the public and the news media (including at conferences and professional meetings) about my scientific research findings, regardless of the level of political contentiousness of the topic.									
**Frequency<br>Row Pct<br>Col Pct**	**CDC**	**EPA**	**FDA**	**NOAA**	**NPS**	**USFWS**	**USGS**	**USDA**	**Energy Agencies**
**1 = Strongly Disagree**	84<br>20.39<br>15.22	68<br>16.5<br>15.74	43<br>10.44<br>13.03	45<br>10.92<br>4.02	29<br>7.04<br>12.89	47<br>11.41<br>13.66	31<br>7.52<br>5.67	36<br><br>12.54	20<br><br>15.63
**2 = Disagree**	138<br>15.92<br>25	138<br>15.92<br>31.94	74<br>8.54<br>22.42	176<br>20.3<br>15.71	53<br>6.11<br>23.56	74<br>8.54<br>21.51	99<br>11.42<br>18.1	73<br><br>25.44	26<br><br>20.31
	203	164	133	411	87	135	181	79	51

**3 = Do Not Agree or Disagree**	13.85<br>36.78	11.19<br>37.96	9.07<br>40.3	28.04<br>36.7	5.93<br>38.67	9.21<br>39.24	12.35<br>33.09	27.53	39.84
**4 = Agree**	83<br>8.73<br>15.04	33<br>3.47<br>7.64	46<br>4.84<br>13.94	369<br>38.8<br>32.95	49<br>5.15<br>21.78	67<br>7.05<br>19.48	200<br>21.03<br>36.56	75<br><br>26.13	18<br><br>14.06
**5 = Strongly Agree**	17<br>7.91<br>3.08	11<br>5.12<br>2.55	15<br>6.98<br>4.55	98<br>45.58<br>8.75	3<br>1.4<br>1.33	12<br>5.58<br>3.49	33<br>15.35<br>6.03	20<br><br>6.97	4<br><br>3.13
**6 = Prefer Not to Disclose**	27<br>23.28<br>4.89	18<br>15.52<br>4.17	19<br>16.38<br>5.76	21<br>18.1<br>1.88	4<br>3.45<br>1.78	9<br>7.76<br>2.62	3<br>2.59<br>0.55	4<br><br>1.39	9<br><br>7.03
**Total**	552	432	330	1120	225	344	547	287	128

Table of Q20 by Agency

Q20 My agency collects the scientific and monitoring information needed to effectively meet its mission.

**Frequency<br>Row Pct<br>Col Pct**	**CDC**	**EPA**	**FDA**	**NOAA**	**NPS**	**USFWS**	**USGS**	**USDA**	**Energy Agencies**
**1 = Always**	97<br>18.69<br>17.48	32<br>6.17<br>7.39	64<br>12.33<br>19.28	130<br>25.05<br>11.59	5<br>0.96<br>2.22	17<br>3.28<br>4.9	89<br>17.15<br>16.24	45<br><br>15.68	20<br><br>15.63
**2 = Frequently**	342<br>15.64<br>61.62	177<br>8.09<br>40.88	167<br>7.64<br>50.3	684<br>31.28<br>60.96	98<br>4.48<br>43.56	108<br>4.94<br>31.12	360<br>16.46<br>65.69	159<br><br>55.40	56<br><br>43.75
**3 = Occasionally**	67<br>8.74<br>12.07	102<br>13.3<br>23.56	56<br>7.3<br>16.87	196<br>25.55<br>17.47	88<br>11.47<br>39.11	136<br>17.73<br>39.19	63<br>8.21<br>11.5	27<br><br>9.41	27<br><br>21.09
**4 = Seldom**	10<br>4.42<br>1.8	49<br>21.68<br>11.32	10<br>4.42<br>3.01	40<br>17.7<br>3.57	29<br>12.83<br>12.89	66<br>29.2<br>19.02	8<br>3.54<br>1.46	6<br><br>2.09	6<br><br>4.69
**5 = Never**	1<br>4.55<br>0.18	6<br>27.27<br>1.39	1<br>4.55<br>0.3	6<br>27.27<br>0.53	0<br>0<br>0	3<br>13.64<br>0.86	1<br>4.55<br>0.18	2<br><br>0.70	3<br><br>2.34
**6 = Don’t Know**	33<br>11.46<br>5.95	58<br>20.14<br>13.39	29<br>10.07<br>8.73	61<br>21.18<br>5.44	5<br>1.74<br>2.22	15<br>5.21<br>4.32	24<br>8.33<br>4.38	48<br><br>16.72	16<br><br>12.50
	5	9	5	5	0	2	3	0	0

**7 = Prefer Not to Disclose**	16.67<br>0.9	30<br>2.08	16.67<br>1.51	16.67<br>0.45	0<br>0	6.67<br>0.58	10<br>0.55	0.00	0.00
**Total**	555	433	332	1122	225	347	548	287	128

Table of Q21 by Agency

Q21 My agency's determinations and actions are consistent with the scientific findings contained in agency documents and reports.

**Frequency**<br>**Row Pct**<br>**Col Pct**	**CDC**	**EPA**	**FDA**	**NOAA**	**NPS**	**USFWS**	**USGS**	**USDA**	**Energy Agencies**
**1 = Always**	130<br>21.35<br>23.59	18<br>2.96<br>4.19	66<br>10.84<br>20.06	132<br>21.67<br>11.78	7<br>1.15<br>3.13	20<br>3.28<br>5.78	145<br>23.81<br>26.61	53<br>18.47	17<br>13.28
**2 = Frequently**	307<br>15.2<br>55.72	157<br>7.77<br>36.51	194<br>9.6<br>58.97	624<br>30.89<br>55.66	104<br>5.15<br>46.43	154<br>7.62<br>44.51	261<br>12.92<br>47.89	129<br>44.95	55<br>42.97
**3 = Occasionally**	43<br>7.73<br>7.8	120<br>21.58<br>27.91	26<br>4.68<br>7.9	128<br>23.02<br>11.42	75<br>13.49<br>33.48	88<br>15.83<br>25.43	27<br>4.86<br>4.95	25<br>8.71	25<br>19.53
**4 = Seldom**	5<br>4.72<br>0.91	35<br>33.02<br>8.14	1<br>0.94<br>0.3	25<br>23.58<br>2.23	11<br>10.38<br>4.91	14<br>13.21<br>4.05	4<br>3.77<br>0.73	6<br>2.09	5<br>3.91
**5 = Never**	0<br>0<br>0	5<br>41.67<br>1.16	1<br>8.33<br>0.3	3<br>25<br>0.27	0<br>0<br>0	0<br>0<br>0	0<br>0<br>0	1<br>0.35	2<br>1.56
**6 = Don't Know**	61<br>9.13<br>11.07	79<br>11.83<br>18.37	31<br>4.64<br>9.42	202<br>30.24<br>18.02	25<br>3.74<br>11.16	65<br>9.73<br>18.79	104<br>15.57<br>19.08	72<br>25.09	24<br>18.75
**7 = Prefer Not to Disclose**	5<br>9.62<br>0.91	16<br>30.77<br>3.72	10<br>19.23<br>3.04	7<br>13.46<br>0.62	2<br>3.85<br>0.89	5<br>9.62<br>1.45	4<br>7.69<br>0.73	1<br>0.35	0<br>0.00
**Total**	551	430	329	1121	224	346	545	287	128

Table of Q22 by Agency

Q22 Expert advice from scientific advisory committees is heeded and incorporated into agency decisions.

**Frequency**<br>**Row Pct**	**CDC**	**EPA**	**FDA**	**NOAA**	**NPS**	**USFWS**	**USGS**	**USDA**	**Energy Agencies**

Col Pct									
**1 = Always**	96<br>30.48<br>17.49	23<br>7.3<br>5.36	44<br>13.97<br>13.41	62<br>19.68<br>5.56	3<br>0.95<br>1.34	13<br>4.13<br>3.77	30<br>9.52<br>5.54	24<br><br>8.33	11<br><br>8.53
**2 = Frequently**	264<br>17.48<br>48.09	101<br>6.69<br>23.54	167<br>11.06<br>50.91	448<br>29.67<br>40.14	64<br>4.24<br>28.57	109<br>7.22<br>31.59	175<br>11.59<br>32.29	109<br><br>37.85	39<br><br>30.23
**3 = Occasionally**	48<br>6.45<br>8.74	99<br>13.31<br>23.08	46<br>6.18<br>14.02	227<br>30.51<br>20.34	80<br>10.75<br>35.71	92<br>12.37<br>26.67	83<br>11.16<br>15.31	38<br><br>13.19	23<br><br>17.83
**4 = Seldom**	11<br>5.02<br>2	53<br>24.2<br>12.35	4<br>1.83<br>1.22	49<br>22.37<br>4.39	20<br>9.13<br>8.93	22<br>10.05<br>6.38	33<br>15.07<br>6.09	11<br><br>3.82	12<br><br>9.30
**5 = Never**	0<br>0<br>0	6<br>20.69<br>1.4	1<br>3.45<br>0.3	3<br>10.34<br>0.27	4<br>13.79<br>1.79	0<br>0<br>0	7<br>24.14<br>1.29	4<br><br>1.39	4<br><br>3.10
**6 = Don't Know**	127<br>10.95<br>23.13	135<br>11.64<br>31.47	60<br>5.17<br>18.29	323<br>27.84<br>28.94	52<br>4.48<br>23.21	107<br>9.22<br>31.01	209<br>18.02<br>38.56	102<br><br>35.42	39<br><br>30.23
**7 = Prefer Not to Disclose**	3<br>8.57<br>0.55	12<br>34.29<br>2.8	6<br>17.14<br>1.83	4<br>11.43<br>0.36	1<br>2.86<br>0.45	2<br>5.71<br>0.58	5<br>14.29<br>0.92	0<br><br>0.00	1<br><br>0.78
**Total**	549	429	328	1116	224	345	542	288	129

Table of Q23 by Agency

Q23 I feel that my scientific work and opinions consistently inform policy decisions.

Frequency<br>Row Pct<br>Col Pct	CDC	EPA	FDA	NOAA	NPS	USFWS	USGS	USDA	Energy Agencies
**1 = Strongly Disagree**	23<br>13.29<br>4.2	26<br>15.03<br>6.02	18<br>10.4<br>5.52	40<br>23.12<br>3.6	11<br>6.36<br>4.93	15<br>8.67<br>4.36	14<br>8.09<br>2.56	13<br><br>4.53	13<br><br>10.16
**2 = Disagree**	64<br>9.55<br>11.68	97<br>14.48<br>22.45	40<br>5.97<br>12.27	166<br>24.78<br>14.94	46<br>6.87<br>20.63	85<br>12.69<br>24.71	88<br>13.13<br>16.09	52<br><br>18.12	28<br><br>21.88
**3 = Do Not Agree or Disagree**	193<br>13.23<br>35.22	158<br>10.83<br>36.57	87<br>5.96<br>26.69	426<br>29.2<br>38.34	90<br>6.17<br>40.36	125<br>8.57<br>36.34	205<br>14.05<br>37.48	113<br><br>39.37	34<br><br>26.56

	CDC	EPA	FDA	NOAA	NPS	USFWS	USGS	USDA	Energy Agencies
**4 = Agree**	219<br>15.77<br>39.96	119<br>8.57<br>27.55	137<br>9.86<br>42.02	397<br>28.58<br>35.73	66<br>4.75<br>29.6	101<br>7.27<br>29.36	199<br>14.33<br>36.38	93<br><br>32.40	41<br><br>32.03
**5 = Strongly Agree**	39<br>15.23<br>7.12	23<br>8.98<br>5.32	35<br>13.67<br>10.74	71<br>27.73<br>6.39	9<br>3.52<br>4.04	14<br>5.47<br>4.07	38<br>14.84<br>6.95	10<br><br>3.48	8<br><br>6.25
**6 = Prefer Not to Disclose**	10<br>16.67<br>1.82	9<br>15<br>2.08	9<br>15<br>2.76	11<br>18.33<br>0.99	1<br>1.67<br>0.45	4<br>6.67<br>1.16	3<br>5<br>0.55	6<br><br>2.09	4<br><br>3.13
**Total**	548	432	326	1111	223	344	547	287	128

Table of Q24 by Agency

Q24 In the past year, I have been excluded from discussions or decisions related to my scientific work that I normally would expect to be a part of.

**Frequency<br>Row Pct<br>Col Pct**	**CDC**	**EPA**	**FDA**	**NOAA**	**NPS**	**USFWS**	**USGS**	**USDA**	**Energy Agencies**
**1 = Strongly Disagree**	90<br>13.68<br>16.48	40<br>6.08<br>9.32	66<br>10.03<br>20.31	213<br>32.37<br>19.15	15<br>2.28<br>6.76	42<br>6.38<br>12.21	91<br>13.83<br>16.64	59<br><br>20.56	23<br><br>17.83
**2 = Disagree**	221<br>13.95<br>40.48	148<br>9.34<br>34.5	127<br>8.02<br>39.08	458<br>28.91<br>41.19	93<br>5.87<br>41.89	126<br>7.95<br>36.63	226<br>14.27<br>41.32	118<br><br>41.11	34<br><br>26.36
**3 = Do Not Agree or Disagree**	119<br>12.23<br>21.79	130<br>13.36<br>30.3	71<br>7.3<br>21.85	262<br>26.93<br>23.56	67<br>6.89<br>30.18	86<br>8.84<br>25	137<br>14.08<br>25.05	68<br><br>23.69	29<br><br>22.48
**4 = Agree**	83<br>15.78<br>15.2	73<br>13.88<br>17.02	41<br>7.79<br>12.62	119<br>22.62<br>10.7	37<br>7.03<br>16.67	65<br>12.36<br>18.9	61<br>11.6<br>11.15	22<br><br>7.67	19<br><br>14.73
**5 = Strongly Agree**	22<br>10.89<br>4.03	28<br>13.86<br>6.53	14<br>6.93<br>4.31	51<br>25.25<br>4.59	9<br>4.46<br>4.05	21<br>10.4<br>6.1	31<br>15.35<br>5.67	12<br><br>4.18	17<br><br>13.18
**6 = Prefer Not to Disclose**	11<br>18.64<br>2.01	10<br>16.95<br>2.33	6<br>10.17<br>1.85	9<br>15.25<br>0.81	1<br>1.69<br>0.45	4<br>6.78<br>1.16	1<br>1.69<br>0.18	8<br><br>2.79	7<br><br>5.43
**Total**	546	429	325	1112	222	344	547	287	129

Table of Q25 by Agency

Q25 I have been pressured or directed by supervisors or senior level officials to NOT attend professional meetings or give public talks on my scientific work.									
**Frequency**<br>**Row Pct**<br>**Col Pct**	**CDC**	**EPA**	**FDA**	**NOAA**	**NPS**	**USFWS**	**USGS**	**USDA**	**Energy Agencies**
**1 = Strongly Disagree**	162<br>14.48<br>29.83	70<br>6.26<br>16.39	94<br>8.4<br>28.92	397<br>35.48<br>35.73	38<br>3.4<br>17.12	63<br>5.63<br>18.37	122<br>10.9<br>22.3	109<br><br>37.98	33<br><br>25.78
**2 = Disagree**	251<br>14.42<br>46.22	186<br>10.68<br>43.56	139<br>7.98<br>42.77	472<br>27.11<br>42.48	102<br>5.86<br>45.95	148<br>8.5<br>43.15	255<br>14.65<br>46.62	117<br><br>40.77	46<br><br>35.94
**3 = Do Not Agree or Disagree**	75<br>9.91<br>13.81	124<br>16.38<br>29.04	59<br>7.79<br>18.15	178<br>23.51<br>16.02	51<br>6.74<br>22.97	98<br>12.95<br>28.57	101<br>13.34<br>18.46	41<br><br>14.29	29<br><br>22.66
**4 = Agree**	25<br>11.21<br>4.6	25<br>11.21<br>5.85	15<br>6.73<br>4.62	43<br>19.28<br>3.87	24<br>10.76<br>10.81	20<br>8.97<br>5.83	47<br>21.08<br>8.59	15<br><br>5.23	8<br><br>6.25
**5 = Strongly Agree**	19<br>20<br>3.5	6<br>6.32<br>1.41	9<br>9.47<br>2.77	13<br>13.68<br>1.17	6<br>6.32<br>2.7	12<br>12.63<br>3.5	18<br>18.95<br>3.29	2<br><br>0.70	7<br><br>5.47
**6 = Prefer Not to Disclose**	11<br>18.64<br>2.03	16<br>27.12<br>3.75	9<br>15.25<br>2.77	8<br>13.56<br>0.72	1<br>1.69<br>0.45	2<br>3.39<br>0.58	4<br>6.78<br>0.73	3<br><br>1.05	5<br><br>3.91
**Total**	543	427	325	1111	222	343	547	287	128

Table of Q26 by Agency									
**Q26 Advisory committees at my agency are constituted by individuals with appropriate expertise and who are able to provide independent scientific advice.**									
**Frequency**<br>**Row Pct**<br>**Col Pct**	**CDC**	**EPA**	**FDA**	**NOAA**	**NPS**	**USFWS**	**USGS**	**USDA**	**Energy Agencies**
**1 = Strongly Disagree**	10<br>6.76<br>1.87	51<br>34.46<br>12.14	11<br>7.43<br>3.42	23<br>15.54<br>2.09	10<br>6.76<br>4.52	15<br>10.14<br>4.41	10<br>6.76<br>1.85	6<br><br>2.14	10<br><br>8.00
**2 = Disagree**	26<br>6.57<br>4.86	97<br>24.49<br>23.1	19<br>4.8<br>5.9	107<br>27.02<br>9.74	33<br>8.33<br>14.93	38<br>9.6<br>11.18	43<br>10.86<br>7.93	20<br><br>7.14	9<br><br>7.20
	169<br>11.09	161<br>10.56	96<br>6.3	433<br>28.41	110<br>7.22	176<br>11.55	224<br>14.7	101	42

**3 = Do Not Agree or Disagree**	31.59	38.33	29.81	39.4	49.77	51.76	41.33	36.07	33.60
**4 = Agree**	222<br>16.36<br>41.5	74<br>5.45<br>17.62	133<br>9.8<br>41.3	405<br>29.85<br>36.85	59<br>4.35<br>26.7	86<br>6.34<br>25.29	196<br>14.44<br>36.16	116<br><br>41.43	45<br><br>36.00
**5 = Strongly Agree**	95<br>21.94<br>17.76	16<br>3.7<br>3.81	56<br>12.93<br>17.39	112<br>25.87<br>10.19	6<br>1.39<br>2.71	18<br>4.16<br>5.29	61<br>14.09<br>11.25	34<br><br>12.14	18<br><br>14.40
**6 = Prefer Not to Disclose**	13<br>14.94<br>2.43	21<br>24.14<br>5	7<br>8.05<br>2.17	19<br>21.84<br>1.73	3<br>3.45<br>1.36	7<br>8.05<br>2.06	8<br>9.2<br>1.48	3<br><br>1.07	1<br><br>0.80
**Total**	535	420	322	1099	221	340	542	280	125

**Table of Q27 by Agency**									
**Q27 In the last year, the expertise on scientific advisory committees at my agency has:**									
**Frequency<br>Row Pct<br>Col Pct**	**CDC**	**EPA**	**FDA**	**NOAA**	**NPS**	**USFWS**	**USGS**	**USDA**	**Energy Agencies**
**1 = Significantly Improved**	8<br>20<br>1.49	5<br>12.5<br>1.18	5<br>12.5<br>1.55	12<br>30<br>1.09	1<br>2.5<br>0.45	1<br>2.5<br>0.29	0<br>0<br>0	2<br><br>0.71	4<br><br>3.23
**2 = Somewhat Improved**	17<br>15.6<br>3.17	6<br>5.5<br>1.42	16<br>14.68<br>4.97	35<br>32.11<br>3.17	4<br>3.67<br>1.81	6<br>5.5<br>1.76	6<br>5.5<br>1.1	6<br><br>2.14	10<br><br>8.06
**3 = No Change**	153<br>15.38<br>28.54	23<br>2.31<br>5.44	119<br>11.96<br>36.96	325<br>32.66<br>29.44	23<br>2.31<br>10.41	62<br>6.23<br>18.18	133<br>13.37<br>24.36	93<br><br>33.10	44<br><br>35.48
**4 = Somewhat Deteriorated**	44<br>9.76<br>8.21	95<br>21.06<br>22.46	26<br>5.76<br>8.07	94<br>20.84<br>8.51	37<br>8.2<br>16.74	58<br>12.86<br>17.01	63<br>13.97<br>11.54	26<br><br>9.25	9<br><br>7.26
**5 = Significantly Deteriorated**	11<br>3.36<br>2.05	163<br>49.85<br>38.53	11<br>3.36<br>3.42	18<br>5.5<br>1.63	43<br>13.15<br>19.46	31<br>9.48<br>9.09	28<br>8.56<br>5.13	14<br><br>4.98	7<br><br>5.65
**6 = Don't Know**	296<br>14.8<br>55.22	123<br>6.15<br>29.08	140<br>7<br>43.48	617<br>30.85<br>55.89	112<br>5.6<br>50.68	181<br>9.05<br>53.08	312<br>15.6<br>57.14	136<br><br>48.40	48<br><br>38.71
**7 = Prefer Not to Disclose**	7<br>18.92	8<br>21.62	5<br>13.51	3<br>8.11	1<br>2.7	2<br>5.41	4<br>10.81	4	2

	CDC	EPA	FDA	NOAA	NPS	USFWS	USGS	USDA	Energy Agencies
	1.31	1.89	1.55	0.27	0.45	0.59	0.73	1.42	1.61
**Total**	536	423	322	1104	221	341	546	281	124

Table of Q28a by Agency

Q28a The level of consideration of political interests hinders the ability of my agency to make science-based decisions.

Frequency<br>Row Pct<br>Col Pct	CDC	EPA	FDA	NOAA	NPS	USFWS	USGS	USDA	Energy Agencies
**1 = Strongly Disagree**	17<br>9.71<br>3.23	6<br>3.43<br>1.42	23<br>13.14<br>7.26	56<br>32<br>5.1	2<br>1.14<br>0.9	8<br>4.57<br>2.35	26<br>14.86<br>4.81	26<br><br>9.25	11<br><br>8.87
**2 = Disagree**	90<br>13.95<br>17.08	17<br>2.64<br>4.02	80<br>12.4<br>25.24	206<br>31.94<br>18.78	16<br>2.48<br>7.24	23<br>3.57<br>6.76	110<br>17.05<br>20.33	59<br><br>21.00	23<br><br>18.55
**3 = Do Not Agree or Disagree**	148<br>13.78<br>28.08	46<br>4.28<br>10.87	98<br>9.12<br>30.91	359<br>33.43<br>32.73	34<br>3.17<br>15.38	69<br>6.42<br>20.29	172<br>16.01<br>31.79	82<br><br>29.18	38<br><br>30.65
**4 = Agree**	172<br>12.88<br>32.64	155<br>11.61<br>36.64	79<br>5.92<br>24.92	373<br>27.94<br>34	109<br>8.16<br>49.32	151<br>11.31<br>44.41	173<br>12.96<br>31.98	84<br><br>29.89	31<br><br>25.00
**5 = Strongly Agree**	83<br>13.34<br>15.75	190<br>30.55<br>44.92	22<br>3.54<br>6.94	89<br>14.31<br>8.11	59<br>9.49<br>26.7	84<br>13.5<br>24.71	50<br>8.04<br>9.24	26<br><br>9.25	17<br><br>13.71
**6 = Prefer Not to Disclose**	17<br>20.99<br>3.23	9<br>11.11<br>2.13	15<br>18.52<br>4.73	14<br>17.28<br>1.28	1<br>1.23<br>0.45	5<br>6.17<br>1.47	10<br>12.35<br>1.85	4<br><br>1.42	4<br><br>3.23
**Total**	527	423	317	1097	221	340	541	281	124

Table of Q28b by Agency

Q28b The level of consideration of business interests hinders the ability of my agency to make science-based decisions.

Frequency<br>Row Pct<br>Col Pct	CDC	EPA	FDA	NOAA	NPS	USFWS	USGS	USDA	Energy Agencies
**1 = Strongly Disagree**	45<br>21.23<br>8.54	10<br>4.72<br>2.37	12<br>5.66<br>3.79	49<br>23.11<br>4.47	2<br>0.94<br>0.91	9<br>4.25<br>2.64	40<br>18.87<br>7.46	24<br><br>8.57	13<br><br>10.48

**2 = Disagree**	138<br>16.83<br>26.19	24<br>2.93<br>5.69	81<br>9.88<br>25.55	231<br>28.17<br>21.08	28<br>3.41<br>12.73	32<br>3.9<br>9.38	147<br>17.93<br>27.43	89<br><br>31.79	29<br><br>23.39
**3 = Do Not Agree or Disagree**	194<br>15.45<br>36.81	58<br>4.62<br>13.74	103<br>8.2<br>32.49	384<br>30.57<br>35.04	64<br>5.1<br>29.09	108<br>8.6<br>31.67	202<br>16.08<br>37.69	83<br><br>29.64	38<br><br>30.65
**4 = Agree**	94<br>8.82<br>17.84	146<br>13.7<br>34.6	85<br>7.97<br>26.81	326<br>30.58<br>29.74	89<br>8.35<br>40.45	119<br>11.16<br>34.9	106<br>9.94<br>19.78	64<br><br>22.86	29<br><br>23.39
**5 = Strongly Agree**	40<br>7.95<br>7.59	171<br>34<br>40.52	29<br>5.77<br>9.15	90<br>17.89<br>8.21	36<br>7.16<br>16.36	70<br>13.92<br>20.53	37<br>7.36<br>6.9	18<br><br>6.43	13<br><br>10.48
**6 = Prefer Not to Disclose**	16<br>23.88<br>3.04	13<br>19.4<br>3.08	7<br>10.45<br>2.21	16<br>23.88<br>1.46	1<br>1.49<br>0.45	3<br>4.48<br>0.88	4<br>5.97<br>0.75	2<br><br>0.71	2<br><br>1.61
**Total**	527	422	317	1096	220	341	536	280	124

**Table of Q28c by Agency**									
**Q28c The level of consideration of non-governmental organization interests hinders the ability of my agency to make science-based decisions.**									
**Frequency<br>Row Pct<br>Col Pct**	**CDC**	**EPA**	**FDA**	**NOAA**	**NPS**	**USFWS**	**USGS**	**USDA**	**Energy Agencies**
**1 = Strongly Disagree**	36<br>15.86<br>6.86	24<br>10.57<br>5.69	11<br>4.85<br>3.47	54<br>23.79<br>4.93	6<br>2.64<br>2.73	9<br>3.96<br>2.64	41<br>18.06<br>7.65	23<br><br>8.21	12<br><br>9.68
**2 = Disagree**	167<br>14.24<br>31.81	101<br>8.61<br>23.93	107<br>9.12<br>33.75	308<br>26.26<br>28.1	57<br>4.86<br>25.91	76<br>6.48<br>22.29	196<br>16.71<br>36.57	103<br><br>36.79	41<br><br>33.06
**3 = Do Not Agree or Disagree**	212<br>12.93<br>40.38	186<br>11.34<br>44.08	107<br>6.52<br>33.75	479<br>29.21<br>43.7	95<br>5.79<br>43.18	151<br>9.21<br>44.28	235<br>14.33<br>43.84	112<br><br>40.00	41<br><br>33.06
**4 = Agree**	82<br>12.31<br>15.62	64<br>9.61<br>15.17	75<br>11.26<br>23.66	210<br>31.53<br>19.16	53<br>7.96<br>24.09	75<br>11.26<br>21.99	45<br>6.76<br>8.4	33<br><br>11.79	22<br><br>17.74
**5 = Strongly Agree**	17<br>11.11<br>3.24	37<br>24.18<br>8.77	9<br>5.88<br>2.84	28<br>18.3<br>2.55	8<br>5.23<br>3.64	27<br>17.65<br>7.92	14<br>9.15<br>2.61	6<br><br>2.14	6<br><br>4.84
	11	10	8	17	1	3	5	3	2

**6 = Prefer Not to Disclose**	17.74<br>2.1	16.13<br>2.37	12.9<br>2.52	27.42<br>1.55	1.61<br>0.45	4.84<br>0.88	8.06<br>0.93	1.07	1.61
**Total**	525	422	317	1096	220	341	536	280	124

Table of Q28d by Agency

Q28d The level of consideration of public opinion hinders the ability of my agency to make science-based decisions.

Frequency<br>Row Pct<br>Col Pct	CDC	EPA	FDA	NOAA	NPS	USFWS	USGS	USDA	Energy Agencies
**1 = Strongly Disagree**	45<br>16.19<br>8.57	40<br>14.39<br>9.46	14<br>5.04<br>4.42	63<br>22.66<br>5.76	12<br>4.32<br>5.45	10<br>3.6<br>2.93	47<br>16.91<br>8.79	23<br>8.21	17<br>13.71
**2 = Disagree**	196<br>13.73<br>37.33	161<br>11.27<br>38.06	115<br>8.05<br>36.28	397<br>27.8<br>36.29	66<br>4.62<br>30	98<br>6.86<br>28.74	242<br>16.95<br>45.23	99<br>35.36	36<br>29.03
**3 = Do Not Agree or Disagree**	191<br>13.9<br>36.38	141<br>10.26<br>33.33	95<br>6.91<br>29.97	415<br>30.2<br>37.93	66<br>4.8<br>30	125<br>9.1<br>36.66	181<br>13.17<br>33.83	97<br>34.64	44<br>35.48
**4 = Agree**	69<br>10.33<br>13.14	56<br>8.38<br>13.24	76<br>11.38<br>23.97	173<br>25.9<br>15.81	65<br>9.73<br>29.55	89<br>13.32<br>26.1	54<br>8.08<br>10.09	54<br>19.29	20<br>16.13
**5 = Strongly Agree**	13<br>11.21<br>2.48	16<br>13.79<br>3.78	8<br>6.9<br>2.52	33<br>28.45<br>3.02	10<br>8.62<br>4.55	17<br>14.66<br>4.99	6<br>5.17<br>1.12	5<br>1.79	5<br>4.03
**6 = Prefer Not to Disclose**	11<br>20<br>2.1	9<br>16.36<br>2.13	9<br>16.36<br>2.84	13<br>23.64<br>1.19	1<br>1.82<br>0.45	2<br>3.64<br>0.59	5<br>9.09<br>0.93	2<br>0.71	2<br>1.61
**Total**	525	423	317	1094	220	341	535	280	124

Table of Q29 by Agency

Q29 The presence of senior decision makers at my agency who come from regulated industry or those with financial interest in regulatory outcomes inappropriately influences the decisions made by the agency.

Frequency<br>Row Pct<br>Col Pct	CDC	EPA	FDA	NOAA	NPS	USFWS	USGS	USDA	Energy Agencies
	56	13	30	73	3	12	63	52	17

**1 = Strongly Disagree**	16.42<br>10.69	3.81<br>3.1	8.8<br>9.55	21.41<br>6.7	0.88<br>1.38	3.52<br>3.54	18.48<br>11.75	18.57	13.82
**2 = Disagree**	120<br>15.44<br>22.9	20<br>2.57<br>4.77	81<br>10.42<br>25.8	212<br>27.28<br>19.45	33<br>4.25<br>15.14	48<br>6.18<br>14.16	148<br>19.05<br>27.61	70<br>25.00	31<br>25.20
**3 = Do Not Agree or Disagree**	216<br>14.65<br>41.22	77<br>5.22<br>18.38	121<br>8.21<br>38.54	471<br>31.95<br>43.21	83<br>5.63<br>38.07	134<br>9.09<br>39.53	203<br>13.77<br>37.87	103<br>36.79	46<br>37.40
**4 = Agree**	72<br>9.34<br>13.74	137<br>17.77<br>32.7	55<br>7.13<br>17.52	230<br>29.83<br>21.1	54<br>7<br>24.77	83<br>10.77<br>24.48	87<br>11.28<br>16.23	38<br>13.57	17<br>13.82
**5 = Strongly Agree**	39<br>8.99<br>7.44	156<br>35.94<br>37.23	15<br>3.46<br>4.78	81<br>18.66<br>7.43	40<br>9.22<br>18.35	54<br>12.44<br>15.93	29<br>6.68<br>5.41	11<br>3.93	10<br>8.13
**6 = Prefer Not to Disclose**	21<br>20<br>4.01	16<br>15.24<br>3.82	12<br>11.43<br>3.82	23<br>21.9<br>2.11	5<br>4.76<br>2.29	8<br>7.62<br>2.36	6<br>5.71<br>1.12	6<br>2.14	2<br>1.63
**Total**	524	419	314	1090	218	339	536	280	123

Table of Q30 by Agency

Q30 Are you able to review, prior to publication, the final drafts of agency communications that are being published under your name and/or that substantially rely on your research?

**Frequency**<br>**Row Pct**<br>**Col Pct**	**CDC**	**EPA**	**FDA**	**NOAA**	**NPS**	**USFWS**	**USGS**	**USDA**	**Energy Agencies**
**1 = Yes**	362<br>16.29<br>68.82	144<br>6.48<br>34.87	173<br>7.79<br>55.27	601<br>27.05<br>55.09	128<br>5.76<br>59.26	142<br>6.39<br>42.9	409<br>18.41<br>75.88	167<br>59.22	53<br>43.09
**2 = No**	19<br>8.44<br>3.61	37<br>16.44<br>8.96	17<br>7.56<br>5.43	67<br>29.78<br>6.14	14<br>6.22<br>6.48	24<br>10.67<br>7.25	20<br>8.89<br>3.71	10<br>3.55	16<br>13.01
**3 = Don't Know**	129<br>9.76<br>24.52	200<br>15.13<br>48.43	106<br>8.02<br>33.87	395<br>29.88<br>36.21	71<br>5.37<br>32.87	155<br>11.72<br>46.83	106<br>8.02<br>19.67	98<br>34.75	47<br>38.21
**4 = Prefer Not to Disclose**	16<br>12.8<br>3.04	32<br>25.6<br>7.75	17<br>13.6<br>5.43	28<br>22.4<br>2.57	3<br>2.4<br>1.39	10<br>8<br>3.02	4<br>3.2<br>0.74	7<br>2.48	7<br>5.69
**Total**	526	413	313	1091	216	331	539	282	123

Table of Q31a by Agency									
**Q31a Are you required to obtain agency pre-approval to communicate with journalists?**									
**Frequency<br>Row Pct<br>Col Pct**	**CDC**	**EPA**	**FDA**	**NOAA**	**NPS**	**USFWS**	**USGS**	**USDA**	**Energy Agencies**
**1 = Yes**	445<br>17.71<br>84.6	358<br>14.25<br>85.04	241<br>9.59<br>76.75	513<br>20.42<br>46.98	134<br>5.33<br>61.47	211<br>8.4<br>62.43	250<br>9.95<br>46.3	221<br><br>78.37	94<br><br>75.81
**2 = No**	9<br>1.36<br>1.71	11<br>1.66<br>2.61	6<br>0.9<br>1.91	344<br>51.89<br>31.5	28<br>4.22<br>12.84	60<br>9.05<br>17.75	174<br>26.24<br>32.22	26<br><br>9.22	1<br><br>0.81
**3 = Don't Know**	64<br>9.41<br>12.17	46<br>6.76<br>10.93	59<br>8.68<br>18.79	216<br>31.76<br>19.78	55<br>8.09<br>25.23	61<br>8.97<br>18.05	110<br>16.18<br>20.37	30<br><br>10.64	27<br><br>21.77
**4 = Prefer Not to Disclose**	8<br>13.33<br>1.52	6<br>10<br>1.43	8<br>13.33<br>2.55	19<br>31.67<br>1.74	1<br>1.67<br>0.46	6<br>10<br>1.78	6<br>10<br>1.11	5<br><br>1.77	2<br><br>1.61
**Total**	526	421	314	1092	218	338	540	282	124

Table of Q31b by Agency									
**Q31b Has this affected your ability to communicate your science externally?**									
**Frequency<br>Row Pct<br>Col Pct**	**CDC**	**EPA**	**FDA**	**NOAA**	**NPS**	**USFWS**	**USGS**	**USDA**	**Energy Agencies**
**1 = Yes**	71<br>16.67<br>15.96	78<br>18.31<br>21.91	38<br>8.92<br>15.77	66<br>15.49<br>12.92	29<br>6.81<br>21.64	52<br>12.21<br>24.64	42<br>9.86<br>16.8	30<br><br>13.64	19<br><br>20.43
**2 = No**	291<br>18.39<br>65.39	182<br>11.5<br>51.12	154<br>9.73<br>63.9	358<br>22.63<br>70.06	71<br>4.49<br>52.99	109<br>6.89<br>51.66	170<br>10.75<br>68	151<br><br>68.64	53<br><br>56.99
**3 = Don't Know**	63<br>14.29<br>14.16	90<br>20.41<br>25.28	42<br>9.52<br>17.43	77<br>17.46<br>15.07	32<br>7.26<br>23.88	49<br>11.11<br>23.22	38<br>8.62<br>15.2	34<br><br>15.45	17<br><br>18.28
**4 = Prefer Not to Disclose**	20<br>35.09<br>4.49	6<br>10.53<br>1.69	7<br>12.28<br>2.9	10<br>17.54<br>1.96	2<br>3.51<br>1.49	1<br>1.75<br>0.47	0<br>0<br>0	5<br><br>2.27	4<br><br>4.30

**Total**	445	356	241	511	134	211	250	220	93

Table of Q32 by Agency

Q32 In the past year, how has your ability to communicate your scientific work to the public and to the media, including the use of social media, changed?

Frequency<br>Row Pct<br>Col Pct	CDC	EPA	FDA	NOAA	NPS	USFWS	USGS	USDA	Energy Agencies
**1 = Significantly Improved**	5<br>14.29<br>0.96	4<br>11.43<br>0.95	3<br>8.57<br>0.96	12<br>34.29<br>1.1	1<br>2.86<br>0.46	2<br>5.71<br>0.59	4<br>11.43<br>0.74	2<br><br>0.71	3<br><br>2.44
**2 = Somewhat Improved**	9<br>5<br>1.72	3<br>1.67<br>0.71	10<br>5.56<br>3.18	107<br>59.44<br>9.83	5<br>2.78<br>2.31	6<br>3.33<br>1.78	31<br>17.22<br>5.75	3<br><br>1.06	3<br><br>2.44
**3 = No Change**	319<br>14.43<br>61.11	152<br>6.88<br>36.19	195<br>8.82<br>62.1	696<br>31.49<br>63.91	101<br>4.57<br>46.76	168<br>7.6<br>49.85	291<br>13.17<br>53.99	176<br><br>62.41	62<br><br>50.41
**4 = Somewhat Deteriorated**	74<br>10.54<br>14.18	117<br>16.67<br>27.86	23<br>3.28<br>7.32	119<br>16.95<br>10.93	68<br>9.69<br>31.48	86<br>12.25<br>25.52	142<br>20.23<br>26.35	48<br><br>17.02	18<br><br>14.63
**5 = Significantly Deteriorated**	24<br>10.3<br>4.6	72<br>30.9<br>17.14	14<br>6.01<br>4.46	29<br>12.45<br>2.66	20<br>8.58<br>9.26	25<br>10.73<br>7.42	29<br>12.45<br>5.38	14<br><br>4.96	9<br><br>7.32
**6 = Don’t Know**	82<br>16.6<br>15.71	60<br>12.15<br>14.29	63<br>12.75<br>20.06	118<br>23.89<br>10.84	21<br>4.25<br>9.72	46<br>9.31<br>13.65	39<br>7.89<br>7.24	37<br><br>13.12	26<br><br>21.14
**7 = Prefer Not to Disclose**	9<br>19.15<br>1.72	12<br>25.53<br>2.86	6<br>12.77<br>1.91	8<br>17.02<br>0.73	0<br>0<br>0	4<br>8.51<br>1.19	3<br>6.38<br>0.56	2<br><br>0.71	2<br><br>1.63
**Total**	522	420	314	1089	216	337	539	282	123

Table of Q33 by Agency

Q33 My agency adheres to its scientific integrity policy (or statement of commitment to scientific integrity).

Frequency<br>Row Pct<br>Col Pct	CDC	EPA	FDA	NOAA	NPS	USFWS	USGS	USDA	Energy Agencies
	17	30	3	25	7	14	13	3	10

	CDC	EPA	FDA	NOAA	NPS	USFWS	USGS	USDA	Energy Agencies
**1 = Strongly Disagree**	13.82<br>3.33	24.39<br>7.19	2.44<br>0.96	20.33<br>2.31	5.69<br>3.26	11.38<br>4.18	10.57<br>2.41	1.06	8.06
**2 = Disagree**	16<br>6.2<br>3.14	70<br>27.13<br>16.79	14<br>5.43<br>4.5	53<br>20.54<br>4.9	22<br>8.53<br>10.23	41<br>15.89<br>12.24	18<br>6.98<br>3.34	16<br><br>5.67	7<br><br>5.65
**3 = Do Not Agree or Disagree**	25<br>6.17<br>4.9	69<br>17.04<br>16.55	36<br>8.89<br>11.58	106<br>26.17<br>9.8	36<br>8.89<br>16.74	52<br>12.84<br>15.52	33<br>8.15<br>6.12	32<br><br>11.35	10<br><br>8.06
**4 = Agree**	234<br>13.87<br>45.88	109<br>6.46<br>26.14	149<br>8.83<br>47.91	518<br>30.71<br>47.87	97<br>5.75<br>45.12	140<br>8.3<br>41.79	251<br>14.88<br>46.57	119<br><br>42.20	46<br><br>37.10
**5 = Strongly Agree**	155<br>19.02<br>30.39	35<br>4.29<br>8.39	60<br>7.36<br>19.29	178<br>21.84<br>16.45	23<br>2.82<br>10.7	37<br>4.54<br>11.04	204<br>25.03<br>37.85	76<br><br>26.95	21<br><br>16.94
**6 = Don't Know**	59<br>10.54<br>11.57	95<br>16.96<br>22.78	45<br>8.04<br>14.47	199<br>35.54<br>18.39	29<br>5.18<br>13.49	50<br>8.93<br>14.93	20<br>3.57<br>3.71	35<br><br>12.41	28<br><br>22.58
**7 = Prefer Not to Disclose**	4<br>16<br>0.78	9<br>36<br>2.16	4<br>16<br>1.29	3<br>12<br>0.28	1<br>4<br>0.47	1<br>4<br>0.3	0<br>0<br>0	1<br><br>0.35	2<br><br>1.61
**Total**	510	417	311	1082	215	335	539	282	124

Table of Q34 by Agency

Q34 I have received adequate training regarding the contents and procedures in my agency's scientific integrity policy (or statement of commitment to scientific integrity).

**Frequency<br>Row Pct<br>Col Pct**	**CDC**	**EPA**	**FDA**	**NOAA**	**NPS**	**USFWS**	**USGS**	**USDA**	**Energy Agencies**
**1 = Strongly Disagree**	19<br>11.45<br>3.73	13<br>7.83<br>3.11	16<br>9.64<br>5.18	64<br>38.55<br>5.92	6<br>3.61<br>2.79	15<br>9.04<br>4.46	3<br>1.81<br>0.56	13<br><br>4.61	18<br><br>14.52
**2 = Disagree**	52<br>7.75<br>10.22	58<br>8.64<br>13.88	56<br>8.35<br>18.12	318<br>47.39<br>29.42	38<br>5.66<br>17.67	66<br>9.84<br>19.64	18<br>2.68<br>3.34	30<br><br>10.64	28<br><br>22.58
**3 = Do Not Agree or Disagree**	76<br>11.11<br>14.93	76<br>11.11<br>18.18	71<br>10.38<br>22.98	271<br>39.62<br>25.07	35<br>5.12<br>16.28	65<br>9.5<br>19.35	26<br>3.8<br>4.82	34<br><br>12.06	21<br><br>16.94
**4 = Agree**	252<br>14.86	206<br>12.15	128<br>7.55	351<br>20.7	113<br>6.66	142<br>8.37	303<br>17.87	132	39

	49.51	49.28	41.42	32.47	52.56	42.26	56.22	46.81	31.45
**5 = Strongly Agree**	109<br>17.67<br>21.41	58<br>9.4<br>13.88	34<br>5.51<br>11	64<br>10.37<br>5.92	22<br>3.57<br>10.23	47<br>7.62<br>13.99	187<br>30.31<br>34.69	71<br><br>25.18	16<br><br>12.90
**6 = Prefer Not to Disclose**	1<br>2.78<br>0.2	7<br>19.44<br>1.67	4<br>11.11<br>1.29	13<br>36.11<br>1.2	1<br>2.78<br>0.47	1<br>2.78<br>0.3	2<br>5.56<br>0.37	2<br><br>0.71	2<br><br>1.61
**Total**	509	418	309	1081	215	336	539	282	124

**Table of Q35 by Agency**									
**Q35 I have been adequately trained on whistleblower rights and protections.**									
**Frequency<br>Row Pct<br>Col Pct**	**CDC**	**EPA**	**FDA**	**NOAA**	**NPS**	**USFWS**	**USGS**	**USDA**	**Energy Agencies**
**1 = Strongly Disagree**	25<br>16.89<br>4.85	14<br>9.46<br>3.34	21<br>14.19<br>6.77	59<br>39.86<br>5.46	5<br>3.38<br>2.33	6<br>4.05<br>1.79	4<br>2.7<br>0.74	11<br><br>3.90	6<br><br>4.88
**2 = Disagree**	74<br>13.33<br>14.37	62<br>11.17<br>14.8	81<br>14.59<br>26.13	209<br>37.66<br>19.33	26<br>4.68<br>12.09	30<br>5.41<br>8.93	23<br>4.14<br>4.27	33<br><br>11.70	19<br><br>15.45
**3 = Do Not Agree or Disagree**	71<br>14.95<br>13.79	58<br>12.21<br>13.84	50<br>10.53<br>16.13	166<br>34.95<br>15.36	14<br>2.95<br>6.51	33<br>6.95<br>9.82	22<br>4.63<br>4.08	40<br><br>14.18	21<br><br>17.07
**4 = Agree**	255<br>13.07<br>49.51	212<br>10.87<br>50.6	126<br>6.46<br>40.65	507<br>25.99<br>46.9	131<br>6.71<br>60.93	200<br>10.25<br>59.52	293<br>15.02<br>54.36	142<br><br>50.35	48<br><br>39.02
**5 = Strongly Agree**	87<br>12.12<br>16.89	68<br>9.47<br>16.23	26<br>3.62<br>8.39	127<br>17.69<br>11.75	39<br>5.43<br>18.14	67<br>9.33<br>19.94	196<br>27.3<br>36.36	56<br><br>19.86	29<br><br>23.58
**6 = Prefer Not to Disclose**	3<br>9.68<br>0.58	5<br>16.13<br>1.19	6<br>19.35<br>1.94	13<br>41.94<br>1.2	0<br>0<br>0	0<br>0<br>0	1<br>3.23<br>0.19	0<br><br>0.00	0<br><br>0.00
**Total**	515	419	310	1081	215	336	539	282	123

**Table of Q36 by Agency**									
**Q36 If I were to obtain knowledge about a scientific integrity issue, I would most likely:**									
**Frequency**	**CDC**	**EPA**	**FDA**	**NOAA**	**NPS**	**USFWS**	**USGS**	**USDA**	

Row Pct<br>Col Pct									Energy Agencies
1 = Be willing to come forward. I trust that the agency would fairly assess and address the issue.	230<br>16.12<br>45.01	80<br>5.61<br>19.18	120<br>8.41<br>38.96	395<br>27.68<br>36.68	48<br>3.36<br>22.33	90<br>6.31<br>27.03	292<br>20.46<br>54.38	91<br><br>32.38	38<br><br>31.40
2 = Be willing to come forward, although I do not trust that the agency would fairly assess and address the issue.	89<br>8.31<br>17.42	136<br>12.7<br>32.61	67<br>6.26<br>21.75	302<br>28.2<br>28.04	94<br>8.78<br>43.72	118<br>11.02<br>35.44	126<br>11.76<br>23.46	101<br><br>35.94	32<br><br>26.45
3 = Not feel comfortable coming forward. I do not trust that the agency would fairly assess and address the issue.	38<br>11.05<br>7.44	80<br>23.26<br>19.18	33<br>9.59<br>10.71	68<br>19.77<br>6.31	30<br>8.72<br>13.95	36<br>10.47<br>10.81	27<br>7.85<br>5.03	19<br><br>6.76	14<br><br>11.57
4 = Not feel comfortable coming forward, although I do trust the agency would fairly assess and address the issue.	30<br>20.55<br>5.87	8<br>5.48<br>1.92	14<br>9.59<br>4.55	43<br>29.45<br>3.99	1<br>0.68<br>0.47	8<br>5.48<br>2.4	20<br>13.7<br>3.72	14<br><br>4.98	6<br><br>4.96
5 = Not feel comfortable coming forward because I would fear suffering retaliation for reporting the violation.	45<br>14.33<br>8.81	50<br>15.92<br>11.99	24<br>7.64<br>7.79	74<br>23.57<br>6.87	18<br>5.73<br>8.37	37<br>11.78<br>11.11	33<br>10.51<br>6.15	16<br><br>5.69	14<br><br>11.57

	CDC	EPA	FDA	NOAA	NPS	USFWS	USGS	USDA	Energy Agencies
**6 = Don't Know**	68<br>14.11<br>13.31	52<br>10.79<br>12.47	42<br>8.71<br>13.64	176<br>36.51<br>16.34	20<br>4.15<br>9.3	36<br>7.47<br>10.81	35<br>7.26<br>6.52	34<br><br>12.10	15<br><br>12.40
**7 = Prefer Not to Disclose**	11<br>14.67<br>2.15	11<br>14.67<br>2.64	8<br>10.67<br>2.6	19<br>25.33<br>1.76	4<br>5.33<br>1.86	8<br>10.67<br>2.4	4<br>5.33<br>0.74	6<br><br>2.14	2<br><br>1.65
**Total**	511	417	308	1077	215	333	537	281	121

Table of Q37 by Agency

[*All agencies, except for FDA, Census Bureau and NHTSA*]

Q37 I have been asked or told to omit the phrase "climate change" from my work.

Frequency<br>Row Pct<br>Col Pct	CDC	EPA	FDA	NOAA	NPS	USFWS	USGS	USDA	Energy Agencies
**1 = Strongly Disagree**	117<br>17.36<br>23.12	37<br>5.49<br>8.87	0<br>0<br>.	296<br>43.92<br>27.48	10<br>1.48<br>4.65	56<br>8.31<br>16.87	69<br>10.24<br>12.87	59<br><br>21.00	30<br><br>24.79
**2 = Disagree**	165<br>13.6<br>32.61	95<br>7.83<br>22.78	0<br>0<br>.	435<br>35.86<br>40.39	53<br>4.37<br>24.65	134<br>11.05<br>40.36	195<br>16.08<br>36.38	108<br><br>38.43	33<br><br>27.27
**3 = Do Not Agree or Disagree**	178<br>20.51<br>35.18	121<br>13.94<br>29.02	0<br>0<br>.	215<br>24.77<br>19.96	49<br>5.65<br>22.79	80<br>9.22<br>24.1	142<br>16.36<br>26.49	63<br><br>22.42	18<br><br>14.88
**4 = Agree**	16<br>3.9<br>3.16	84<br>20.49<br>20.14	0<br>0<br>.	88<br>21.46<br>8.17	62<br>15.12<br>28.84	38<br>9.27<br>11.45	83<br>20.24<br>15.49	27<br><br>9.61	23<br><br>19.01
**5 = Strongly Agree**	9<br>4.43<br>1.78	63<br>31.03<br>15.11	0<br>0<br>.	20<br>9.85<br>1.86	38<br>18.72<br>17.67	21<br>10.34<br>6.33	29<br>14.29<br>5.41	18<br><br>6.41	12<br><br>9.92
**6 = Prefer Not to Disclose**	21<br>22.11<br>4.15	17<br>17.89<br>4.08	0<br>0<br>.	23<br>24.21<br>2.14	3<br>3.16<br>1.4	3<br>3.16<br>0.9	18<br>18.95<br>3.36	6<br><br>2.14	5<br><br>4.13
**Total**	506	417	0	1077	215	332	536	281	121

Table of Q38a by Agency

[*All agencies, except for FDA, Census Bureau and NHTSA*]

Q38a I have been asked or told to avoid work on climate change.

Frequency<br>Row Pct<br>Col Pct	CDC	EPA	FDA	NOAA	NPS	USFWS	USGS	USDA	Energy Agencies
1 = Strongly Disagree	119<br>16.69<br>23.52	35<br>4.91<br>8.41	0<br>0<br>.	321<br>45.02<br>29.81	23<br>3.23<br>10.7	58<br>8.13<br>17.47	64<br>8.98<br>12.01	64<br><br>22.78	30<br><br>24.59
2 = Disagree	165<br>12.39<br>32.61	96<br>7.21<br>23.08	0<br>0<br>.	482<br>36.19<br>44.75	90<br>6.76<br>41.86	146<br>10.96<br>43.98	219<br>16.44<br>41.09	105<br><br>37.37	32<br><br>26.23
3 = Do Not Agree or Disagree	184<br>18.53<br>36.36	153<br>15.41<br>36.78	0<br>0<br>.	220<br>22.16<br>20.43	63<br>6.34<br>29.3	102<br>10.27<br>30.72	163<br>16.41<br>30.58	80<br><br>28.47	31<br><br>25.41
4 = Agree	16<br>6.3<br>3.16	79<br>31.1<br>18.99	0<br>0<br>.	29<br>11.42<br>2.69	25<br>9.84<br>11.63	20<br>7.87<br>6.02	59<br>23.23<br>11.07	18<br><br>6.41	16<br><br>13.11
5 = Strongly Agree	6<br>6.25<br>1.19	37<br>38.54<br>8.89	0<br>0<br>.	9<br>9.38<br>0.84	11<br>11.46<br>5.12	5<br>5.21<br>1.51	16<br>16.67<br>3	8<br><br>2.85	9<br><br>7.38
6 = Prefer Not to Disclose	16<br>22.22<br>3.16	16<br>22.22<br>3.85	0<br>0<br>.	16<br>22.22<br>1.49	3<br>4.17<br>1.4	1<br>1.39<br>0.3	12<br>16.67<br>2.25	6<br><br>2.14	4<br><br>3.28
Total	506	416	0	1077	215	332	533	281	122

Table of Q38b by Agency

[*All agencies, except for FDA, Census Bureau and NHTSA*]

Q38b This has adversely impacted my effectiveness at my job in my agency.

Frequency<br>Row Pct<br>Col Pct	CDC	EPA	FDA	NOAA	NPS	USFWS	USGS	USDA	Energy Agencies
1 = Strongly Disagree	0<br>0<br>0	1<br>25<br>0.86	0<br>0<br>.	0<br>0<br>0	0<br>0<br>0	0<br>0<br>0	3<br>75<br>4	0<br><br>0.00	0<br><br>0.00
2 = Disagree	5<br>10.87<br>22.73	13<br>28.26<br>11.21	0<br>0<br>.	4<br>8.7<br>10.53	2<br>4.35<br>5.56	3<br>6.52<br>12	13<br>28.26<br>17.33	3<br><br>11.54	4<br><br>16.00
3 = Do Not Agree or Disagree	7<br>7.45<br>31.82	30<br>31.91<br>25.86	0<br>0<br>.	10<br>10.64<br>26.32	16<br>17.02<br>44.44	5<br>5.32<br>20	19<br>20.21<br>25.33	6<br><br>23.08	4<br><br>16.00

**4 = Agree**	6<br>4.41<br>27.27	49<br>36.03<br>42.24	0<br>0<br>.	17<br>12.5<br>44.74	9<br>6.62<br>25	11<br>8.09<br>44	26<br>19.12<br>34.67	11<br><br>42.31	12<br><br>48.00
**5 = Strongly Agree**	3<br>4.41<br>13.64	22<br>32.35<br>18.97	0<br>0<br>.	7<br>10.29<br>18.42	9<br>13.24<br>25	6<br>8.82<br>24	14<br>20.59<br>18.67	6<br><br>23.08	5<br><br>20.00
**6 = Prefer Not to Disclose**	1<br>50<br>4.55	1<br>50<br>0.86	0<br>0<br>.	0<br>0<br>0	0<br>0<br>0	0<br>0<br>0	0<br>0<br>0	0<br><br>0.00	0<br><br>0.00
**Total**	22	116	0	38	36	25	75	26	25

**Table of Q39 by Agency**									
**[*All agencies, except for FDA, Census Bureau and NHTSA*]**									
**Q39 I have avoided working on climate change or using the phrase "climate change," though I was not explicitly told to avoid them.**									
**Frequency<br>Row Pct<br>Col Pct**	**CDC**	**EPA**	**FDA**	**NOAA**	**NPS**	**USFWS**	**USGS**	**USDA**	**Energy Agencies**
**1 = Strongly Disagree**	117<br>17.49<br>23.17	35<br>5.23<br>8.41	0<br>0<br>.	283<br>42.3<br>26.3	26<br>3.89<br>12.09	70<br>10.46<br>21.08	57<br>8.52<br>10.69	51<br><br>18.28	32<br><br>26.89
**2 = Disagree**	143<br>13.11<br>28.32	99<br>9.07<br>23.8	0<br>0<br>.	378<br>34.65<br>35.13	83<br>7.61<br>38.6	120<br>11<br>36.14	156<br>14.3<br>29.27	86<br><br>30.82	27<br><br>22.69
**3 = Do Not Agree or Disagree**	200<br>21.51<br>39.6	138<br>14.84<br>33.17	0<br>0<br>.	218<br>23.44<br>20.26	50<br>5.38<br>23.26	86<br>9.25<br>25.9	145<br>15.59<br>27.2	74<br><br>26.52	26<br><br>21.85
**4 = Agree**	20<br>3.44<br>3.96	100<br>17.21<br>24.04	0<br>0<br>.	162<br>27.88<br>15.06	46<br>7.92<br>21.4	50<br>8.61<br>15.06	142<br>24.44<br>26.64	43<br><br>15.41	24<br><br>20.17
**5 = Strongly Agree**	7<br>6.25<br>1.39	24<br>21.43<br>5.77	0<br>0<br>.	17<br>15.18<br>1.58	9<br>8.04<br>4.19	5<br>4.46<br>1.51	27<br>24.11<br>5.07	20<br><br>7.17	7<br><br>5.88
**6 = Prefer Not to Disclose**	18<br>25.35<br>3.56	20<br>28.17<br>4.81	0<br>0<br>.	18<br>25.35<br>1.67	1<br>1.41<br>0.47	1<br>1.41<br>0.3	6<br>8.45<br>1.13	5<br><br>1.79	3<br><br>2.52
**Total**	505	416	0	1076	215	332	533	279	119

Table of Q43a by Agency								
[*USDA only: ARS / ERS / NIFA / NASS*]								
Q43a My agency has received input or guidance on its scientific work or other information about cross-departmental scientific coordination from the USDA's Office of the Chief Scientist (OCS) during the past year.								
**Frequency<br>Row Pct<br>Col Pct**	**CDC**	**EPA**	**FDA**	**NOAA**	**NPS**	**USFWS**	**USGS**	**USDA**
**1 = Strongly Disagree**	0<br>0<br>.	0<br>0<br>.	0<br>0<br>.	0<br>0<br>.	0<br>0<br>.	0<br>0<br>.	0<br>0<br>.	10<br><br>4.10
**2 = Disagree**	0<br>0<br>.	0<br>0<br>.	0<br>0<br>.	0<br>0<br>.	0<br>0<br>.	0<br>0<br>.	0<br>0<br>.	35<br><br>14.34
**3 = Do Not Agree or Disagree**	0<br>0<br>.	0<br>0<br>.	0<br>0<br>.	0<br>0<br>.	0<br>0<br>.	0<br>0<br>.	0<br>0<br>.	146<br><br>59.84
**4 = Agree**	0<br>0<br>.	0<br>0<br>.	0<br>0<br>.	0<br>0<br>.	0<br>0<br>.	0<br>0<br>.	0<br>0<br>.	38<br><br>15.57
**5 = Strongly Agree**	0<br>0<br>.	0<br>0<br>.	0<br>0<br>.	0<br>0<br>.	0<br>0<br>.	0<br>0<br>.	0<br>0<br>.	3<br><br>1.23
**6 = Prefer Not to Disclose**	0<br>0<br>.	0<br>0<br>.	0<br>0<br>.	0<br>0<br>.	0<br>0<br>.	0<br>0<br>.	0<br>0<br>.	12<br><br>4.92
**Total**	0	0	0	0	0	0	0	244

Table of Q43b by Agency								
[*USDA only: ARS / ERS / NIFA / NASS*]								
Q43b This lack of input/feedback about cross-departmental scientific coordination is a departure from previous OCS practice.								
**Frequency<br>Row Pct<br>Col Pct**	**CDC**	**EPA**	**FDA**	**NOAA**	**NPS**	**USFWS**	**USGS**	**USDA**
**1 = Strongly Disagree**	0<br>0<br>.	0<br>0<br>.	0<br>0<br>.	0<br>0<br>.	0<br>0<br>.	0<br>0<br>.	0<br>0<br>.	2<br><br>4.55
**2 = Disagree**	0	0	0	0	0	0	0	13

	0<br>.	0<br>.	0<br>.	0<br>.	0<br>.	0<br>.	0<br>.	29.55
**3 = Do Not Agree or Disagree**	0<br>0<br>.	0<br>0<br>.	0<br>0<br>.	0<br>0<br>.	0<br>0<br>.	0<br>0<br>.	0<br>0<br>.	13<br>29.55
**4 = Agree**	0<br>0<br>.	0<br>0<br>.	0<br>0<br>.	0<br>0<br>.	0<br>0<br>.	0<br>0<br>.	0<br>0<br>.	10<br>22.73
**5 = Strongly Agree**	0<br>0<br>.	0<br>0<br>.	0<br>0<br>.	0<br>0<br>.	0<br>0<br>.	0<br>0<br>.	0<br>0<br>.	5<br>11.36
**6 = Prefer Not to Disclose**	0<br>0<br>.	0<br>0<br>.	0<br>0<br>.	0<br>0<br>.	0<br>0<br>.	0<br>0<br>.	0<br>0<br>.	1<br>2.27
**Total**	0	0	0	0	0	0	0	44

Table of Q44a by Agency

[*USDA only: ARS / ERS / NIFA / NASS*]

Q44a Over the past year, the OCS has convened the USDA Science Council about as regularly as in previous years.

**Frequency<br>Row Pct<br>Col Pct**	**CDC**	**EPA**	**FDA**	**NOAA**	**NPS**	**USFWS**	**USGS**	**USDA**
**1 = Strongly Disagree**	0<br>0<br>.	0<br>0<br>.	0<br>0<br>.	0<br>0<br>.	0<br>0<br>.	0<br>0<br>.	0<br>0<br>.	4<br>1.67
**2 = Disagree**	0<br>0<br>.	0<br>0<br>.	0<br>0<br>.	0<br>0<br>.	0<br>0<br>.	0<br>0<br>.	0<br>0<br>.	10<br>4.17
**3 = Do Not Agree or Disagree**	0<br>0<br>.	0<br>0<br>.	0<br>0<br>.	0<br>0<br>.	0<br>0<br>.	0<br>0<br>.	0<br>0<br>.	208<br>86.67
**4 = Agree**	0<br>0<br>.	0<br>0<br>.	0<br>0<br>.	0<br>0<br>.	0<br>0<br>.	0<br>0<br>.	0<br>0<br>.	8<br>3.33
**6 = Prefer Not to Disclose**	0<br>0<br>.	0<br>0<br>.	0<br>0<br>.	0<br>0<br>.	0<br>0<br>.	0<br>0<br>.	0<br>0<br>.	10<br>4.17

Total	0	0	0	0	0	0	0	240

Table of Q44b by Agency

[*USDA only: ARS / ERS / NIFA / NASS*]

Q44b This has hampered cross-departmental scientific coordination and collaboration and made it more difficult for my agency to fulfill its science-based mission.

Frequency<br>Row Pct<br>Col Pct	CDC	EPA	FDA	NOAA	NPS	USFWS	USGS	USDA
2 = Disagree	0<br>0<br>.	0<br>0<br>.	0<br>0<br>.	0<br>0<br>.	0<br>0<br>.	0<br>0<br>.	0<br>0<br>.	2<br><br>14.29
3 = Do Not Agree or Disagree	0<br>0<br>.	0<br>0<br>.	0<br>0<br>.	0<br>0<br>.	0<br>0<br>.	0<br>0<br>.	0<br>0<br>.	6<br><br>42.86
4 = Agree	0<br>0<br>.	0<br>0<br>.	0<br>0<br>.	0<br>0<br>.	0<br>0<br>.	0<br>0<br>.	0<br>0<br>.	4<br><br>28.57
5 = Strongly Agree	0<br>0<br>.	0<br>0<br>.	0<br>0<br>.	0<br>0<br>.	0<br>0<br>.	0<br>0<br>.	0<br>0<br>.	2<br><br>14.29
Total	0	0	0	0	0	0	0	14

Table of Q50 by Agency

Q50 What is your current grade level?

Frequency<br>Row Pct<br>Col Pct	CDC	EPA	FDA	NOAA	NPS	USFWS	USGS	USDA	Energy Agencies
1 = GS-11 or lower	51<br>9.27<br>10.02	25<br>4.55<br>5.98	8<br>1.45<br>2.61	103<br>18.73<br>9.58	110<br>20<br>51.16	115<br>20.91<br>34.53	89<br>16.18<br>16.67	32<br><br>11.47	5<br><br>4.20
2 = GS-12 and above	337<br>11.86<br>66.21	374<br>13.16<br>89.47	254<br>8.94<br>82.74	759<br>26.72<br>70.6	104<br>3.66<br>48.37	213<br>7.5<br>63.96	427<br>15.03<br>79.96	222<br><br>79.57	91<br><br>76.47
	42<br>76.36	0<br>0	11<br>20	2<br>3.64	0<br>0	0<br>0	0<br>0	0	0

**3 = Commissioned Corps**	8.25	0	3.58	0.19	0	0	0	0.00	0.00
**4 = Student**	2<br>25<br>0.39	0<br>0<br>0	2<br>25<br>0.65	4<br>50<br>0.37	0<br>0<br>0	0<br>0<br>0	0<br>0<br>0	0<br><br>0.00	0<br><br>0.00
**5 = Fellow**	21<br>55.26<br>4.13	1<br>2.63<br>0.24	6<br>15.79<br>1.95	1<br>2.63<br>0.09	0<br>0<br>0	0<br>0<br>0	1<br>2.63<br>0.19	8<br><br>2.87	8<br><br>6.72
**6 = Visiting Scientist**	0<br>0<br>0	0<br>0<br>0	0<br>0<br>0	5<br>100<br>0.47	0<br>0<br>0	0<br>0<br>0	0<br>0<br>0	0<br><br>0.00	0<br><br>0.00
**7 = Contractor**	34<br>14.91<br>6.68	0<br>0<br>0	11<br>4.82<br>3.58	168<br>73.68<br>15.63	0<br>0<br>0	0<br>0<br>0	7<br>3.07<br>1.31	7<br><br>2.51	7<br><br>0.06
**8 = Other**	10<br>24.39<br>1.96	4<br>9.76<br>0.96	4<br>9.76<br>1.3	15<br>36.59<br>1.4	0<br>0<br>0	0<br>0<br>0	6<br>14.63<br>1.12	1<br><br>0.36	0<br><br>0.00
**9 = Prefer Not to Disclose**	12<br>14.46<br>2.36	14<br>16.87<br>3.35	11<br>13.25<br>3.58	18<br>21.69<br>1.67	1<br>1.2<br>0.47	5<br>6.02<br>1.5	4<br>4.82<br>0.75	9<br><br>3.23	8<br><br>6.72
**Total**	509	418	307	1075	215	333	534	279	119

Table of Q51 by Agency

Q51 How long have you been working at your agency?

**Frequency<br>Row Pct<br>Col Pct**	**CDC**	**EPA**	**FDA**	**NOAA**	**NPS**	**USFWS**	**USGS**	**USDA**	**Energy Agencies**
**1 = Less than 3 years**	103<br>18.33<br>20.24	61<br>10.85<br>14.59	60<br>10.68<br>19.54	122<br>21.71<br>11.33	23<br>4.09<br>10.7	49<br>8.72<br>14.76	57<br>10.14<br>10.67	45<br><br>16.13	30<br><br>25.42
**2 = 3-5 years**	58<br>15.3<br>11.39	23<br>6.07<br>5.5	51<br>13.46<br>16.61	90<br>23.75<br>8.36	18<br>4.75<br>8.37	30<br>7.92<br>9.04	46<br>12.14<br>8.61	27<br><br>9.68	31<br><br>26.27
**3 = 6-10 years**	89<br>13.03<br>17.49	67<br>9.81<br>16.03	67<br>9.81<br>21.82	194<br>28.4<br>18.01	55<br>8.05<br>25.58	59<br>8.64<br>17.77	80<br>11.71<br>14.98	34<br><br>12.19	32<br><br>27.12
**4 = 11-15 years**	69<br>15.2	34<br>7.49	33<br>7.27	161<br>35.46	34<br>7.49	34<br>7.49	49<br>10.79	27	8

	CDC	EPA	FDA	NOAA	NPS	USFWS	USGS	USDA	Energy Agencies
	13.56	8.13	10.75	14.95	15.81	10.24	9.18	9.68	6.78
**5 = More than 15 years**	180<br>10.51<br>35.36	221<br>12.91<br>52.87	90<br>5.26<br>29.32	501<br>29.26<br>46.52	84<br>4.91<br>39.07	157<br>9.17<br>47.29	297<br>17.35<br>55.62	143<br><br>51.25	14<br><br>11.86
**6 = Prefer Not to Disclose**	10<br>16.95<br>1.96	12<br>20.34<br>2.87	6<br>10.17<br>1.95	9<br>15.25<br>0.84	1<br>1.69<br>0.47	3<br>5.08<br>0.9	5<br>8.47<br>0.94	3<br><br>1.08	3<br><br>2.54
**Total**	509	418	307	1077	215	332	534	279	118

Table of Q52 by Agency

Q52 Have you ever worked for regulated industry or a group representing them?

Frequency<br>Row Pct<br>Col Pct	CDC	EPA	FDA	NOAA	NPS	USFWS	USGS	USDA	Energy Agencies
**1 = Yes**	37<br>9.02<br>7.27	107<br>26.1<br>25.6	81<br>19.76<br>26.47	58<br>14.15<br>5.39	19<br>4.63<br>8.84	33<br>8.05<br>9.91	35<br>8.54<br>6.55	16<br><br>5.73	20<br><br>16.95
**2 = No**	467<br>13.74<br>91.75	305<br>8.98<br>72.97	220<br>6.47<br>71.9	1005<br>29.58<br>93.4	195<br>5.74<br>90.7	300<br>8.83<br>90.09	496<br>14.6<br>92.88	260<br><br>93.19	97<br><br>82.20
**3 = Prefer Not to Disclose**	5<br>12.82<br>0.98	6<br>15.38<br>1.44	5<br>12.82<br>1.63	13<br>33.33<br>1.21	1<br>2.56<br>0.47	0<br>0<br>0	3<br>7.69<br>0.56	3<br><br>1.08	1<br><br>0.85
**Total**	509	418	306	1076	215	333	534	279	118

Table of Q53 by Agency

Q53 What is the highest level of education you have completed?

Frequency<br>Row Pct<br>Col Pct	CDC	EPA	FDA	NOAA	NPS	USFWS	USGS	USDA	Energy Agencies
**1 = Bachelor's Degree**	29<br>5.12<br>5.7	75<br>13.25<br>17.99	31<br>5.48<br>10.16	251<br>44.35<br>23.31	33<br>5.83<br>15.35	59<br>10.42<br>17.72	46<br>8.13<br>8.61	12<br><br>4.30	18<br><br>15.25
**2 = Master's Degree**	150<br>10.77<br>29.47	195<br>14<br>46.76	47<br>3.37<br>15.41	408<br>29.29<br>37.88	112<br>8.04<br>52.09	191<br>13.71<br>57.36	165<br>11.84<br>30.9	40<br><br>14.34	59<br><br>50.00
**3 = PhD**	235	128	177	395	66	72	316	217	34

	14.15<br>46.17	7.71<br>30.7	10.66<br>58.03	23.78<br>36.68	3.97<br>30.7	4.33<br>21.62	19.02<br>59.18	77.78	28.81
**4 = MD**	70<br>63.06<br>13.75	1<br>0.9<br>0.24	34<br>30.63<br>11.15	3<br>2.7<br>0.28	0<br>0<br>0	1<br>0.9<br>0.3	2<br>1.8<br>0.37	0<br>0.00	0<br>0.00
**5 = JD**	3<br>15.79<br>0.59	7<br>36.84<br>1.68	0<br>0<br>0	2<br>10.53<br>0.19	1<br>5.26<br>0.47	3<br>15.79<br>0.9	1<br>5.26<br>0.19	0<br>0.00	2<br>1.69
**6 = DVM**	7<br>38.89<br>1.38	0<br>0<br>0	5<br>27.78<br>1.64	0<br>0<br>0	0<br>0<br>0	3<br>16.67<br>0.9	0<br>0<br>0	3<br>1.08	0<br>0.00
**7 = Other**	4<br>21.05<br>0.79	1<br>5.26<br>0.24	6<br>31.58<br>1.97	6<br>31.58<br>0.56	2<br>10.53<br>0.93	0<br>0<br>0	0<br>0<br>0	0<br>0.00	0<br>0.00
**8 = Prefer Not to Disclose**	11<br>18.33<br>2.16	10<br>16.67<br>2.4	5<br>8.33<br>1.64	12<br>20<br>1.11	1<br>1.67<br>0.47	4<br>6.67<br>1.2	4<br>6.67<br>0.75	7<br>2.51	5<br>4.24
**Total**	509	417	305	1077	215	333	534	279	118

Table of Q54_1 by Agency

Q54_1 White, Caucasian

**Frequency<br>Row Pct<br>Col Pct**	**CDC**	**EPA**	**FDA**	**NOAA**	**NPS**	**USFWS**	**USGS**	**USDA**	**Energy Agencies**
**1 = Selected**	338<br>11<br>100	328<br>10.68<br>100	193<br>6.28<br>100	906<br>29.49<br>100	190<br>6.18<br>100	285<br>9.28<br>100	479<br>15.59<br>100	227<br>100.00	80<br>100.00
**Total**	338	328	193	906	190	285	479	227	80

Table of Q54_2 by Agency

Q54_2 Hispanic or Latino/a

**Frequency<br>Row Pct<br>Col Pct**	**CDC**	**EPA**	**FDA**	**NOAA**	**NPS**	**USFWS**	**USGS**	**USDA**	**Energy Agencies**
**1 = Selected**	24<br>19.05<br>100	18<br>14.29<br>100	16<br>12.7<br>100	18<br>14.29<br>100	8<br>6.35<br>100	13<br>10.32<br>100	14<br>11.11<br>100	8<br>100.00	3<br>100.00

Total	24	18	16	18	8	13	14	8	3

Table of Q54_3 by Agency									
Q54_3 Black or African American									
Frequency<br>Row Pct<br>Col Pct	CDC	EPA	FDA	NOAA	NPS	USFWS	USGS	USDA	Energy Agencies
1 = Selected	58<br>49.57<br>100	8<br>6.84<br>100	15<br>12.82<br>100	14<br>11.97<br>100	0<br>0<br>.	7<br>5.98<br>100	3<br>2.56<br>100	3<br><br>100.00	7<br><br>100.00
Total	58	8	15	14	0	7	3	3	7

Table of Q54_4 by Agency									
Q54_4 Native American or American Indian									
Frequency<br>Row Pct<br>Col Pct	CDC	EPA	FDA	NOAA	NPS	USFWS	USGS	USDA	Energy Agencies
1 = Selected	2<br>4.65<br>100	13<br>30.23<br>100	1<br>2.33<br>100	11<br>25.58<br>100	3<br>6.98<br>100	8<br>18.6<br>100	2<br>4.65<br>100	1<br><br>100.00	1<br><br>100.00
Total	2	13	1	11	3	8	2	1	1

Table of Q54_5 by Agency									
Q54_5 Asian / Pacific Islander									
Frequency<br>Row Pct<br>Col Pct	CDC	EPA	FDA	NOAA	NPS	USFWS	USGS	USDA	Energy Agencies
1 = Selected	41<br>21.69<br>100	16<br>8.47<br>100	40<br>21.16<br>100	48<br>25.4<br>100	2<br>1.06<br>100	8<br>4.23<br>100	10<br>5.29<br>100	11<br><br>100.00	8<br><br>100.00
Total	41	16	40	48	2	8	10	11	8

Table of Q54_6 by Agency									
Q54_6 Other									
Frequency	CDC	EPA	FDA	NOAA	NPS	USFWS	USGS	USDA	

Frequency<br>Row Pct<br>Col Pct									Energy Agencies
1 = Selected	8<br>15.38<br>100	10<br>19.23<br>100	4<br>7.69<br>100	13<br>25<br>100	2<br>3.85<br>100	6<br>11.54<br>100	5<br>9.62<br>100	1<br><br>100.00	1<br><br>100.00
Total	8	10	4	13	2	6	5	1	1

Table of Q54_7 by Agency									
Q54_7 Prefer not to disclose									
Frequency<br>Row Pct<br>Col Pct	CDC	EPA	FDA	NOAA	NPS	USFWS	USGS	USDA	Energy Agencies
1 = Selected	50<br>13.62<br>100	50<br>13.62<br>100	47<br>12.81<br>100	91<br>24.8<br>100	15<br>4.09<br>100	22<br>5.99<br>100	35<br>9.54<br>100	31<br><br>100.00	21<br><br>100.00
Total	50	50	47	91	15	22	35	31	21

Table of Q55 by Agency									
Q55 What is your gender?									
Frequency<br>Row Pct<br>Col Pct	CDC	EPA	FDA	NOAA	NPS	USFWS	USGS	USDA	Energy Agencies
1 = Male	187<br>8.82<br>36.81	203<br>9.58<br>48.8	156<br>7.36<br>51.15	676<br>31.89<br>62.88	112<br>5.28<br>52.09	185<br>8.73<br>55.56	351<br>16.56<br>65.73	161<br><br>57.71	60<br><br>51.28
2 = Female	280<br>19.54<br>55.12	180<br>12.56<br>43.27	118<br>8.23<br>38.69	330<br>23.03<br>30.7	88<br>6.14<br>40.93	126<br>8.79<br>37.84	161<br>11.24<br>30.15	87<br><br>31.18	36<br><br>30.77
3 = Nonbinary	3<br>20<br>0.59	2<br>13.33<br>0.48	0<br>0<br>0	2<br>13.33<br>0.19	2<br>13.33<br>0.93	1<br>6.67<br>0.3	2<br>13.33<br>0.37	0<br><br>0.00	1<br><br>0.85
4 = Prefer Not to Disclose	38<br>13.87<br>7.48	31<br>11.31<br>7.45	31<br>11.31<br>10.16	67<br>24.45<br>6.23	13<br>4.74<br>6.05	21<br>7.66<br>6.31	20<br>7.3<br>3.75	31<br><br>11.11	20<br><br>17.09
Total	508	416	305	1075	215	333	534	279	117
